# Identification and function of a novel human memory-like NK cell population expressing CD160 in melioidosis

**DOI:** 10.1016/j.isci.2023.107234

**Published:** 2023-06-28

**Authors:** Anucha Preechanukul, Barbara Kronsteiner, Natnaree Saiprom, Kitilak Rochaikun, Boonthanom Moonmueangsan, Rungnapa Phunpang, Orawan Ottiwet, Yuphin Kongphrai, Soonthon Wapee, Kesinee Chotivanich, Chumpol Morakot, Rachan Janon, Susanna J. Dunachie, Narisara Chantratita

**Affiliations:** 1 Department of Microbiology and Immunology, Faculty of Tropical Medicine, Mahidol University, Bangkok, Thailand; 2 Peter Medawar Building for Pathogen Research, Nuffield Department of Clinical Medicine, University of Oxford, Oxford, UK; 3 NDM Centre for Global Health Research, Nuffield Department of Clinical Medicine, University of Oxford, Oxford, UK; 4 Department of Medical Technology and Clinical Pathology, Mukdahan Hospital, Mukdahan, Thailand; 5 Department of Clinical Tropical Medicine, Faculty of Tropical Medicine, Mahidol University, Bangkok, Thailand; 6 Mahidol-Oxford Tropical Medicine Research Unit, Mahidol University, Bangkok, Thailand; 7 Department of Medicine, Mukdahan Hospital, Mukdahan, Thailand; 8 Oxford University Hospitals NHS Foundation Trust, Oxford, UK

**Keywords:** Immunology, Immunity, Microbiology, Cell biology

## Abstract

NK cells are endowed with immunological memory to a range of pathogens but the development of NK cell memory in bacterial infections remains elusive. Here, we establish an assay inducing memory-like NK cell response to *Burkholderia pseudomallei*, the causative agent of the severe bacterial disease called melioidosis, and explore NK cell memory in a melioidosis patient cohort. We show that NK cells require bacteria-primed monocytes to acquire memory-like properties, demonstrated by bacteria-specific responses, features that strongly associate with CD160 expression. Induction of this memory-like NK cell is partly dependent on CD160 and IL-12R. Importantly, CD160 expression identifies memory-like NK cells in a cohort of recovered melioidosis patients with heightened responses maintained at least 3 months post hospital admission and reduced numbers of this cell population independently correlate with recurrent melioidosis. These newly identified memory-like NK cells are a promising target for future vaccine design and for monitoring protection against infection.

## Highlights

• CD160 is a newly identified marker to track memory-like NK cells in melioidosis
• Induction of *B. pseudomallei*-specific memory-like NK cells is dependent of monocytes
• CD160^+^ NK cells from recovered melioidosis patients exhibited memory-like responses to BP
• Memory-like NK cell responses were reduced in individuals with recurrent melioidosis

## Graphical abstract

**Figure:**
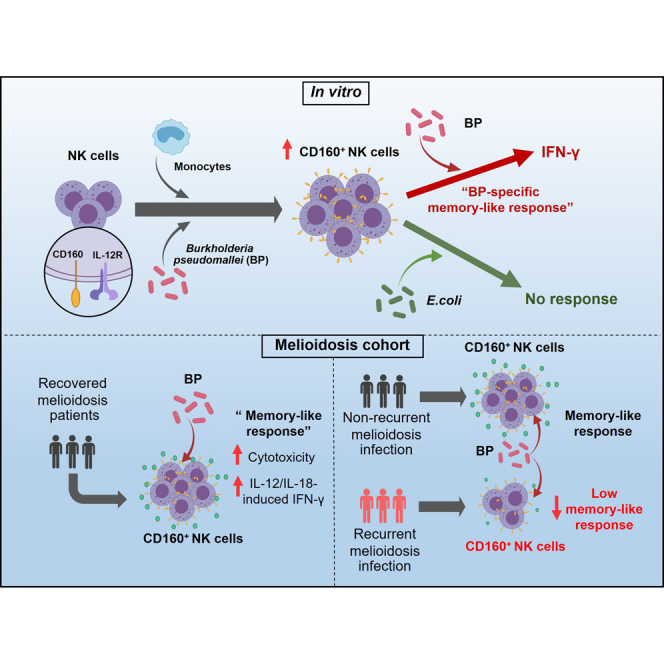

## Introduction

The hallmark features of adaptive immunity are memory T and B cells recognizing previously encountered antigens and mounting a secondary response that is quantitatively and qualitatively different from the primary response. The development of immunological memory is the cornerstone of long-term protective immunity elicited by any successful vaccination against infectious diseases.^1^ Recently, compelling new evidence on the adaptive-like features of innate immune cells has challenged this dogma. Innate immune cells, including monocytes, macrophages, and natural killer (NK) cells, are able to develop immunological memory through epigenetic re-programing upon primary exposure to microbial antigens.^2^^,^^3^ NK cells are a subset of innate lymphoid cells specialized for immune defense against tumors and intracellular infection.^4^ Unlike innate immune memory in phagocytic cells—which exhibit non-specific responses to secondary stimulation with either hypo- or hyper-responsiveness, NK cells are capable of both antigen-specific memory responses and antigen-unspecific memory-like responses upon re-exposure, which do not involve somatically rearranged antigen receptors.^5^^,^^6^^,^^7^ Pathogen-responsive NK cells acquire properties of antigen-specific memory to murine cytomegalovirus (MCMV) infection^8^ and viral antigens.^9^^,^^10^ A growing body of research suggests that NK cells have the potential to mount memory-like responses to a wide range of viral pathogens, including MCMV, human CMV (HCMV), vaccinia virus, Epstein-Barr virus, varicella-zoster virus, and HIV,^8^^,^^11^^,^^12^^,^^13^^,^^14^^,^^15^ and even eukaryotic pathogens.^16^

Evidence of NK cell memory following bacterial infection is still sparse with *Mycobacterium tuberculosis*^17^^,^^18^ and *Ehrlichia muris*^19^ being the only bacterial pathogens explored so far. In a murine model of Bacillus Calmette-Guerin (BCG) immunization, NK cell-derived interferon-γ (IFN-γ) production and proliferation occurs in response to a subsequent challenge with *M. tuberculosis*.^18^ Patients with tuberculosis (TB) show accumulation of memory-like NK cells in the pleural fluid, with this sub-population producing more IL-22 and IFN-γ when co-cultured with BCG-infected monocytes.^17^

NK cells are critical for the early control of intracellular bacterial infection^20^ and have been identified as an immune correlate of protection in acute melioidosis,^21^ a neglected tropical disease caused by the soil-dwelling Gram-negative bacterium *Burkholderia pseudomallei* (BP). BP is prevalent in Southeast Asia and Northern Australia, and is under-reported globally with an estimated 165,000 human cases and 89,000 deaths annually in tropical regions worldwide.^22^^,^^23^ Melioidosis affects vulnerable individuals and type 2 diabetes is the most common risk factor.^23^ The disease poses a serious health threat with hospitalized case fatality exceeding 40% in some tropical countries, compounded by limited therapeutics and lack of a vaccine.^24^ A deeper understanding of NK cells in melioidosis and induction of NK cell memory to BP will inform vaccine design and further illuminate NK memory in bacterial infection.

Here, we investigate the development of NK cell memory in response to BP *in vitro* and in a Thai melioidosis cohort. We establish an assay to generate memory-like NK cells *in vitro* and show that NK cells acquire cell-intrinsic memory-like properties through priming with BP-primed THP-1 cells. These memory-like NK cells upregulate IFN-γ production in response to IL-12 and IL-18 stimulation, display bacteria-specific polyfunctional cytokine responses, and increased killing of intracellular BP upon re-challenge which strongly correlate with CD160 expression. Blocking CD160 and IL-12 receptors on NK cells partially prevents acquisition of memory-like properties. We observe an elevated frequency of CD160-expressing NK cells with memory-like responses in recovered melioidosis patients. To our knowledge, this study is the first report demonstrating the induction of memory-like NK cells in melioidosis and identifying CD160 as a novel marker of BP-specific memory-like NK cells to monitor these cells in future clinical studies.

## Results

### BP-primed monocytes potentiate IFN-γ secretion from NK-92 MI cells

**Figure 1:**
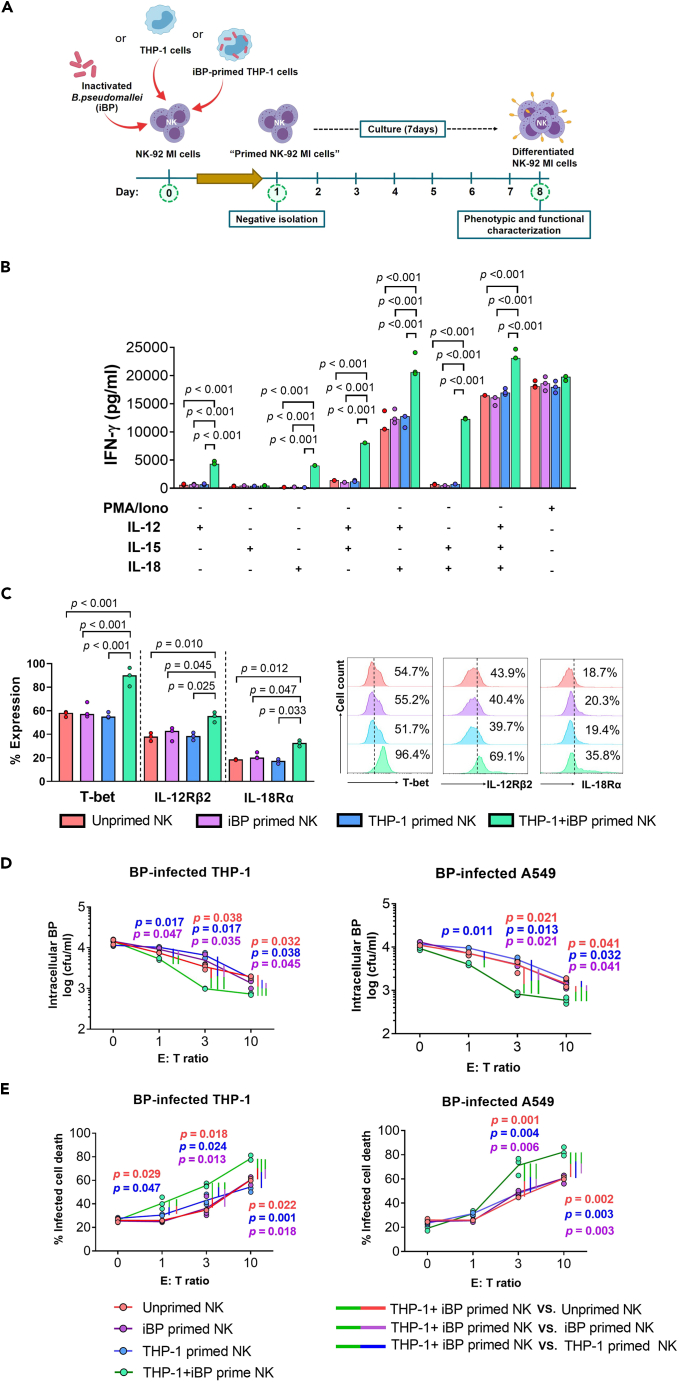
THP-1 cells primed with inactivated *B. pseudomallei* induce phenotypic and functional changes in NK cells (A) Experimental workflow of the NK cell memory assay (created with BioRender.com): NK-92 MI cells were primed with inactivated (heat-killed) BP pre-stimulated THP-1 cells (THP-1 + iBP), unstimulated THP-1 cells, iBP alone or left unprimed overnight. Primed NK-92 MI cells were subsequently purified by negative magnetic enrichment. Unprimed and primed NK cells were cultured for 7 days followed by phenotypic and functional characterization by flow cytometry. (B) IFN-γ levels in supernatants of unprimed or primed NK cells (7 days post priming) in response to 18 h stimulation with IL-12, IL-15, IL-18, or combinations thereof presented in bar graphs. (C) Expression of transcription factor T-bet, IL-12Rβ2, and IL-18Rɑ on unprimed or primed NK cells (7 days post priming) in percentage are shown in bar graphs and representative histograms. (D) Log of viable intracellular bacteria (cfu/mL) after incubation of BP-infected A549 or THP-1 cells with unprimed or primed NK-92 MI cells (7 days post priming) at effector (E) cell: target (T) cell ratios of 0, 1, 3, and 10. (E) Percentage of infected cell death after incubation of BP-infected A549 or THP-1 cells with unprimed or primed NK-92 MI cells (7 days post priming) at E:T ratios of 0, 1, 3, and 10. Dual colored vertical lines between data points indicate which groups were compared for statistical analysis and matching p values are color coded accordingly. Three independent experiments were performed with three technical replicates each. The medians of the technical replicates were used for statistical testing and graphical presentation. Statistical differences were calculated using Kruskal-Wallis, followed by with Dunn’s test with the Benjamini-Hochberg method for multiple comparison. A p value of <0.05 is considered statistically significant.

We first sought to evaluate whether priming with inactivated *B. pseudomallei* (iBP) can induce functional changes akin to the induction of memory-like properties in human NK cells. We chose the human NK-92-derived IL-2 independent cell line (NK-92 MI) to establish a novel NK cell assay for the induction of memory-like features *in vitro* (Figure 1A). We first evaluated whether BP-mediated priming potentiated NK-92 MI cell-derived IFN-γ secretion when stimulated with IL-12, IL-15, IL-18, or combinations thereof. All of these cytokines have been previously reported to induce varying degrees of IFN-γ secretion from NK cells^25^ and memory-like NK cells are known to show potentiated secretion.^26^

When co-cultured with iBP-primed THP-1 cells (THP-1+iBP) at MOI 100, NK-92 MI cells exhibited significantly higher IFN-γ secretion in response to IL-12 and IL-18, respectively (Figure 1B). In contrast, NK cells primed with iBP did not show alterations in cytokine secretion as compared to the unprimed control (Figure 1B). The presence of both IL-12 and IL-18 either with or without IL-15 resulted in a dramatic increase in IFN-γ secretion in all NK cell conditions when compared to NK cells only treated with a single cytokine. Even though differences between groups were marginal in comparison to what was observed in the presence of IL-12 and IL-18 alone, THP-1-primed NK cells still exhibited the highest IFN-γ levels (Figure 1B).

We next established whether priming of THP-1 cells with iBP induced cytokine secretion akin to activated THP-1 cells. Indeed, iBP at MOI 100 significantly increased secretion of IL-12, IL-15, and IL-18 by THP-1 cells compared to lower MOIs (4 and 20), which failed to upregulate cytokine secretion (Figure S1A). In line with this, NK cells primed with THP-1+iBP at a lower MOI did not show an increase in IFN-γ secretion in response to IL-12, IL-15, IL-18, or combinations thereof (Figure S1B). Furthermore, priming with THP-1+iBP significantly increased the frequency and expression levels (median fluorescence intensity, MFI) of T-bet, IL-12Rβ2, and IL-18Rɑ on NK-92 MI cells compared to the control (p < 0.05 for all comparisons) (Figure 1C) indicating that heightened cytokine responsiveness of primed NK-92 MI cells is dependent on the presence of activated THP-1 cells.

We further demonstrate the suitability of this assay to induce memory-like NK cells in response to other bacterial antigens by priming NK cells with inactivated *E*. *coli* (iEC) in the same manner as described for BP. Indeed, NK cells primed with THP-1+iEC showed heightened IFN-γ secretion upon cytokine stimulation compared to unprimed NK cells or those primed with THP-1 or iEC alone (Figure S2) demonstrating the suitability of this *in vitro* assay to study memory-like functions of NK cells in response to bacteria.

### Memory-like NK cells induce enhanced killing of intracellular BP

Another feature of memory-like NK cells is their superior ability to kill target cells.^27^ Thus, we next set up a killing assay with primed NK cells and BP-infected non-phagocytic and phagocytic cells (Figure 1D) to investigate whether BP-primed NK cells eliminate intracellular BP in infected cells more efficiently. NK-92 MI cells, which were left unprimed or primed with iBP, THP-1 cells, or THP-1+iBP, were used as effector (E) cells. A549 and THP-1 cells representative of non-phagocytic and phagocytic host cells were infected with live BP and served as the target (T) cells. Incubation of BP-infected A549 and THP-1 cells for 2 h with increasing numbers of unprimed and primed NK cells (E:T ratios of 1, 3, and 10) reduced the viability of BP, as compared to infected cultures without NK cells (E:T ratio of 0) (Figure 1D). THP-1+iBP-primed NK cells were more potent at killing of intracellular bacteria and both infected THP-1 and A549 cells at E:T ratios of 3 and 10, compared with unprimed, iBP-primed, and THP-1-primed NK cells (p < 0.05 for all comparisons) (Figures 1D and 1E).

### BP-primed NK cells mount pathogen-specific memory-like responses by increasing polyfunctionality and killing

In order to assess whether priming of NK cells with iBP-primed monocytes leads to memory-like responses to BP, we assessed polyfunctional responses in NK cells after re-stimulation with iBP as well as cross-reactive (inactivated *B. thailandensis* E264, iBT) and unrelated bacterial stimuli (inactivated *E. coli* DH5α, iEC) using intracellular flow cytometry staining for the cytotoxic molecules granzyme B (GzmB) and perforin (Pfr), the degranulation marker CD107a and IFN-γ.

**Figure 2:**
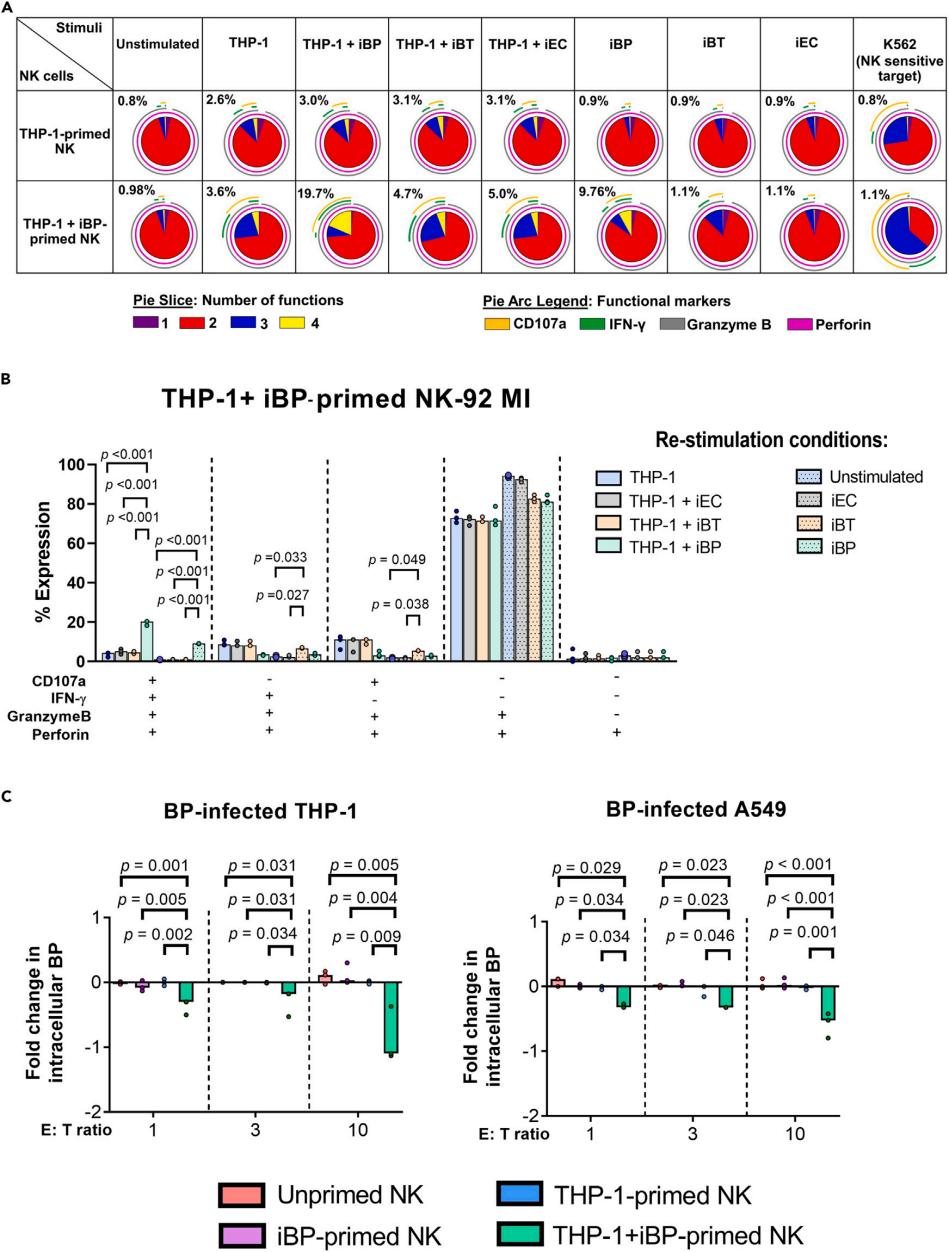
THP-1+iBP-primed NK-92 MI cells exhibit memory-like responses by enhancing polyfunctionality and killing of intracellular BP upon re-stimulation NK-92 MI cells, which were unprimed or primed with heat-killed BP-primed THP-1 cells (THP-1+iBP, MOI 100), unstimulated THP-1, or iBP alone (MOI of 100) for one day. NK cells were negatively purified and then cultured for 7 days. Unprimed or primed NK cells were then stimulated with different antigenic stimuli including inactivated (heat-killed) *B. pseudomallei* (iBP), *B. thailandensis* (iBT) or *E. coli* (iEC), THP-1 cells primed with inactivated bacteria (THP-1+iBP, THP-1+iBT, THP-1+iEC), unprimed THP-1 cells or K-562 cells for 18 h. (A) Pie charts are shown comparing polyfunctionality of unprimed and primed NK cells in response to stimuli. Pie arcs represent proportions of NK cell responses comprising CD107a (orange arcs), IFN-γ (green arcs), granzyme B (gray arcs), and perforin (pink arcs). Pie slices represent the percentage of NK cells co-expressing 1 (violet), 2 (red), 3 (blue), or 4 (yellow) functional markers. The percentage of NK cells with concurrent expression of 4 functions is given above each pie chart. Data were summarized from three-independent experiments, which were performed with three technical replicates each. (B) The percentage of THP-1+iBP-primed NK cells co-expressing CD107a, IFN-γ, granzyme B, and perforin in response to different stimuli. (C) Fold change of reduction in intracellular BP after incubation of BP-infected A549 or THP-1 cells with unprimed or primed NK-92 MI cells, calculated by log [(cfu/mL in the condition with iBP re-stimulated NK cells)/(cfu/mL in the condition with unstimulated NK cells)]. Three independent experiments were performed with three technical replicates each. The medians of the technical replicates were used for statistical testing and graphical presentation. Statistical differences were calculated using Kruskal-Wallis, followed by with Dunn’s method with the Benjamini-Hochberg method for multiple comparison. A p value of <0.05 is considered statistically significant.

The vast majority of THP-1+iBP-primed NK-92 MI cells (>80%) expressed two functional markers (GzmB^+^and Pfr^+^) (Figure 2A). Upon re-stimulation with THP-1+iBP, NK-92 MI cells primed with THP-1+iBP significantly increased their polyfunctional response compared to cells primed with THP-1 alone. The proportion of cells co-expressing four functional markers ranged from 14.2% to 25.3% of the total population and this was due to an augmentation in degranulation and IFN-γ production. Importantly, re-stimulation with iBP alone was able to induce a strong polyfunctional response albeit slightly less diverse than what was observed in the THP-1+iBP re-stimulation condition. THP-1+iBP-primed NK-92 MI cells also increased the proportion of degranulation as well as IFN-γ production in response to cross-reactive species (iBT) but not unrelated stimuli (iEC, K-592 cells) (Figures 2A, 2B, and S3B). Re-stimulation with THP-1+iBP and iBP only induced the enrichment of THP-1+iBP-primed NK-92 MI cells expressing four functional markers, compared to unprimed, iBP-primed, and THP-1-primed NK-92 MI cells. Of note, these re-stimulation conditions gave the unique pattern of functional response in THP-1+iBP-primed NK-92 MI cells, where the functional response pattern was different from THP-1 alone, THP-1+iEC, and THP-1+iBT re-stimulation (Figures 2A, 2B, and S3B). This indicates that THP-1+iBP-primed NK-92 MI cells exhibit memory-like response to BP specifically.

We observed no functional alterations in NK-92 MI cells primed with iBP in the absence of THP-1 cells in response to any of the tested re-stimulation conditions (Figure S3A). Expression levels of GzmB and Pfr were significantly increased in NK-92 MI cells primed with THP-1+iBP, irrespective of the stimuli used for re-stimulation, compared with THP-1-primed NK cells (p < 0.01 all both comparisons) (Figure S3C).

When re-stimulated with iBP-primed, THP-1+iBP-primed NK-92 MI cells had more than 2-fold higher capacity to induce elimination of intracellular BP in both infected A549 and THP-1 cells at E:T ratios of 1, 3, and 10, as compared to unprimed, THP-1-primed, and iBP-primed NK cells (p < 0.01) (Figure 2C).

Taken together, the results demonstrate that NK cells can be induced to mount BP-specific memory-like responses *in vitro*.

### Alteration in phenotypic characteristics of BP-specific memory-like NK cell

We next assessed whether these memory-like functions can be related to specific phenotypic characteristics. In order to do so, we first determined the surface expression of activation markers (CD25, CD69), ontogenesis markers (CD56, CD161), chemokine receptors (CCR7, CXCR3), and NK cell receptors (CD160, NKp30, NKp44, NKp46, NKG2A, and NKG2D) on primed and unprimed NK cells.

**Figure 3:**
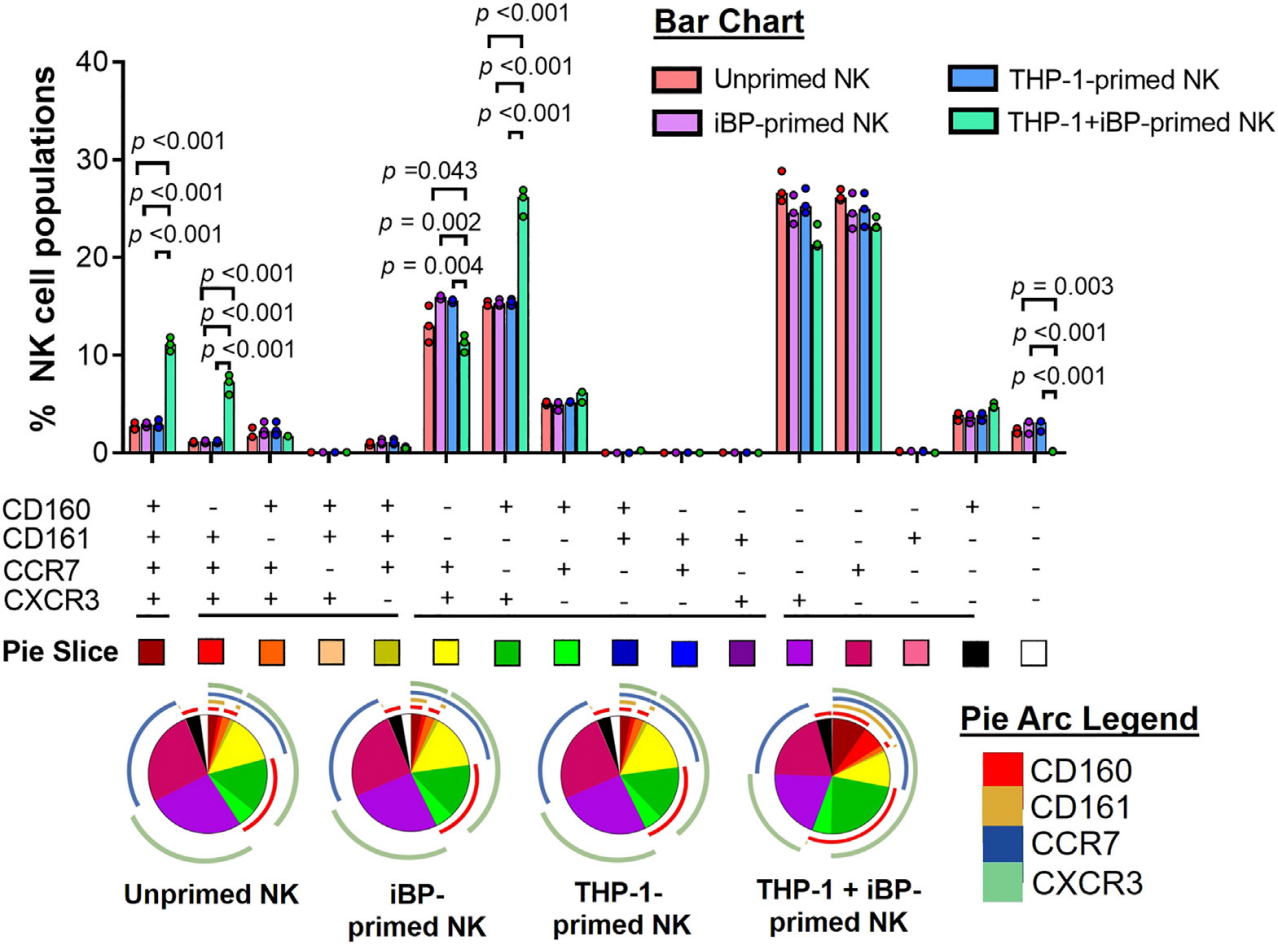
Alteration in phenotypic markers in THP-1+iBP-primed NK-92 MI cells NK-92 MI cells remained untreated or were primed with THP-1 cells previously primed with inactivated (heat-killed) BP (THP-1+iBP, MOI of 100), unstimulated THP-1, or iBP alone (MOI 100) for 24 h and subsequently magnetically enriched and cultured for 7 days. Expression of CD160, CD161, CCR7, and CXCR3 was analyzed using flow cytometry and co-expression patterns are depicted in bar and pie charts. Differently colored pie slices represent the subsets of NK cells expressing a given combination of surface markers and pie arcs represent the individual markers: CD160 (red), CD161 (mustard yellow), CCR7 (blue), CXCR3 (green). Three independent experiments were performed with three technical replicates each. The medians of the technical replicates were used for statistical testing and graphical presentation. Statistical differences were calculated using Kruskal-Wallis test, followed by with Dunn’s test with the Benjamini-Hochberg method for multiple comparison. A p value of <0.05 is considered statistically significant.

Expression of CD160, CD161, CCR7, and CXCR3 (relative frequency and MFI) as well as the MFI of NKp44 and NKp46 was significantly increased on NK cells primed with THP-1+iBP in comparison to THP-1 alone (all p < 0.05) while the other markers remained unchanged. There was no change in expression of markers on iBP-primed NK cells compared to unprimed cells (Figures 3 and S3D). Subsequent combinatorial analysis of CD160, CD161, CCR7, and CXCR3 expression revealed significant enrichment of three NK cell populations upon THP-1+iBP priming compared to THP-1 alone; one subset expressing all four markers, one lacking CD160 expression, and one characterized by CD160 and CXCR3 co-expression (Figure 3).

In order to explore the possible relationship between NK cell sub-populations and functional characteristics, we assessed the association between those three cell subsets as well as CD160 expression alone with NK cell functions including degranulation, IFN-γ production, and NK cell-mediated killing of intracellular bacteria under different priming conditions. Due to low sample size, formal correlation analysis could not be performed on this dataset. However, the frequency of NK cells with a specific surface marker profile was compared against the respective functional parameters (Figures S4A and S4B). In all cases, heighted function in memory-like NK cells was associated with the highest frequency of NK cells expressing CD160 in that sample, highlighting its role as a potential phenotypic marker of BP-specific memory-like NK cells.

### CD160 and IL-12 receptors partly contribute to acquisition of memory-like NK cell properties

**Figure 4:**
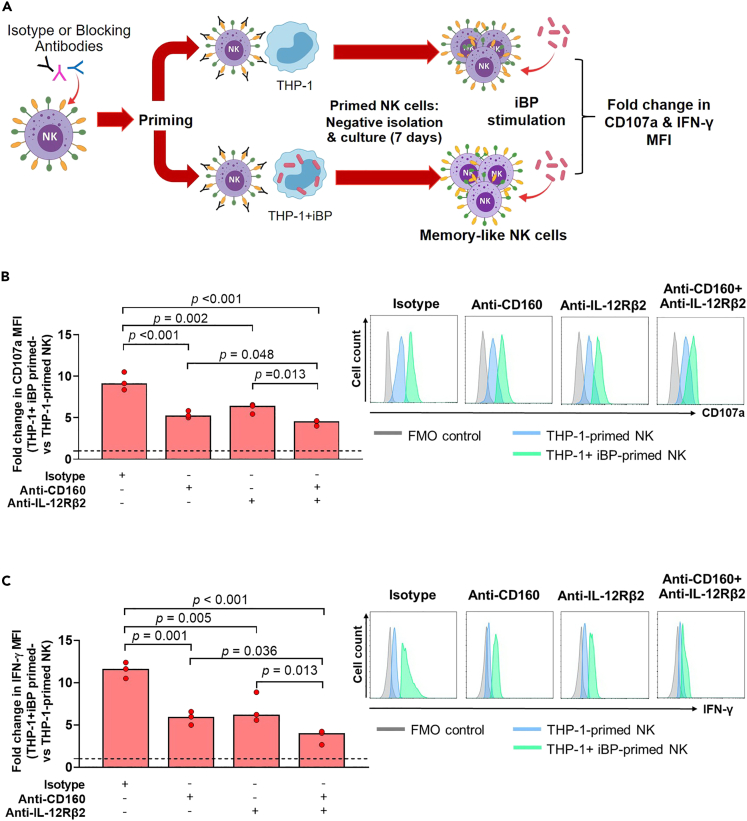
CD160 and IL-12 receptor are partly required for generation of NK cells with memory-like functions (A) NK-92 MI cells were treated with mAbs against CD160 and IL-12Rβ2. Following priming, NK cells were stimulated with inactivated (heat killed) BP (iBP) and degranulation (CD107a) as well as IFN-γ production were measured by flow cytometry. Schematic created with BioRender.com. The fold change of (B) CD107a and (C) IFN-γ expression (median fluorescence intensity, MFI) upon iBP stimulation of THP-1+iBP-primed versus THP-1-primed NK cells. The horizontal dashed line on each graph represents a fold change of 1, where expression levels on THP-1+iBP- and THP-1-primed NK-92 MI cells is equal. Representative histograms are shown for (B) CD107a and (C) IFN-γ expression on THP-1+iBP-primed (green) and THP-1-primed NK cells (blue) treated with isotype or different combinations of mAbs. A fluorescent minus one control is shown in gray. Three independent experiments were performed with three technical replicates each. The medians of the technical replicates were used for statistical testing and graphical presentation. Statistical differences were calculated using Kruskal-Wallis test, followed by with Dunn’s method with the Benjamini-Hochberg method for multiple comparison. A p value of <0.05 is considered statistically significant.

Recent studies indicate that NK cells can directly recognize bacteria, bacteria-infected cells, and MHC class I through activating receptors,^28^ leading to the possibility of bacteria-induced NK cell differentiation into memory-like NK cells. NK cells can also acquire memory-like properties following brief stimulation with IL-12, IL-15, and IL-18.^29^ Based on our results demonstrating increased expression of CD160 and cytokine receptors in NK cells primed with THP-1+iBP and correlation with memory-like responses, we next investigated whether a panel of NK receptors potentially contribute to the generation of memory-like NK cell responses. We used monoclonal antibodies (mAbs) against the following selected receptors on NK cells: 1) Cytokine receptors (IL-12, IL-15, and IL-18 receptors) involved with memory-like NK cell development^26^ and 2) Activating receptors including CD160, together with NKp44 and NKp46—which can directly recognize bacteria and bacteria-infected cells.^28^^,^^30^ NK-92 MI cells were treated with mAbs against those receptors and then primed with THP-1+iBP or THP-1 alone. Following priming and subsequent culture for 7 days, NK-92 MI cells were re-stimulated with iBP and degranulation and IFN-γ production were measured (Figure 4A). Blocking CD160 and IL-12Rβ2 on NK cells was sufficient to induce a significant reduction in the fold change of CD107a and IFN-γ expression upon iBP re-stimulation of THP-1+iBP-primed relative to THP-1-primed NK cells (Figures 4B and 4C). Blocking any of the other receptors either alone or in combination with the above did not result in further inhibition of functional responses of NK-92 MI cells (Figures S4C and S4D). These results indicate that the acquisition of memory-like features of NK cells through priming with BP-stimulated monocytes is partially dependent on CD160 and IL-12Rβ2.

### Memory-like function in primary NK cells is associated with CD160 expression

**Figure 5:**
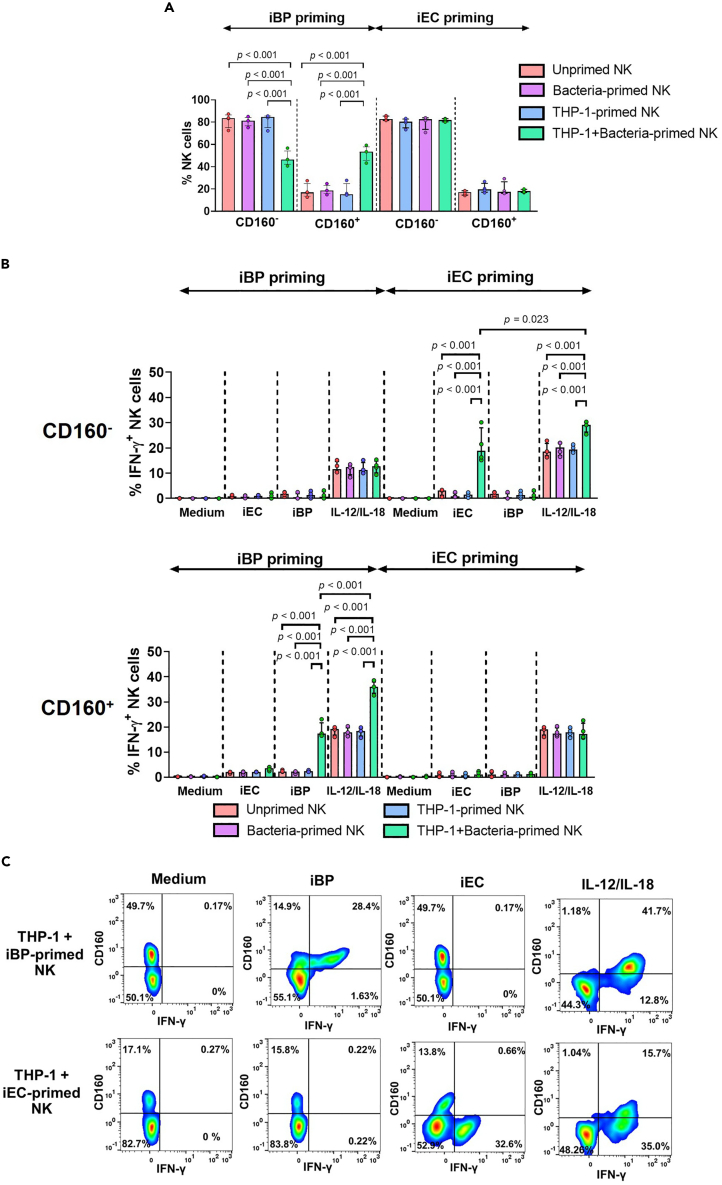
Function of primary BP-induced memory-like NK cells is associated with a subset of CD160-expressing NK cells Primary NK cells isolated from healthy donors (n = 4) were subjected to the established *in vitro* NK cell memory assay (Figure 1A). NK cells were primed with inactivated bacteria (iBP or iEC), THP-1, THP-1+iBP, or THP-1+iEC or left unprimed. (A) The proportion of CD160^-^ and CD160^+^ NK cells was determined by flow cytometry 7 days after priming. (B) Percentage of IFN-γ expression in the CD160^-^ and CD160^+^ subsets of primed NK cells cultured for 7 days and stimulated with iEC, iBP, IL-12+IL-18 or left unstimulated (medium) overnight. Four healthy donors were measured in three independent experiments and the median of the technical replicates was calculated for each donor. Data are presented in bar graphs showing individual donors, median and interquartile range. Statistical analysis was performed using Kruskal-Wallis test, followed by with Dunn’s method with the Benjamini-Hochberg method for multiple comparison, and only p values for statistically significant (p < 0.05) comparisons are shown on the graphs. (C) Representative flow cytometry density plots highlight differences in CD160 and IFN-γ co-expression in THP-1+iBP- and THP+iEC-primed memory-like NK cells in response to different stimuli as described above.

Having shown that THP-1+iBP priming initiates the generation of memory-like features in NK-92 MI cells, we next validated the induction of BP-specific memory-like function using primary NK cells obtained from healthy donors. In accordance with our results using NK-92 MI cells, THP-1+iBP priming induced the upregulation of CD160 on human primary NK cells when compared with unprimed, iBP-primed, and THP-1-primed NK cells (p < 0.001 for all comparisons) (Figure 5A). Re-stimulation with iBP induced IFN-γ production in THP-1+iBP-primed NK cells but not unprimed and other primed conditions and this was specifically associated with the expression of CD160 on NK cells. Stratification of NK cells into CD160^+^ and CD160^-^ subsets showed that heightened IFN-γ secretion upon BP-specific re-stimulation and IL-12/IL-18 stimulation was exclusively detected in the CD160^+^ subset (p < 0.0001 for all comparisons) (Figures 5B and 5C). In contrast, THP-1+iBP priming had no effect on the percentage of IFN-γ-producing NK cells in the CD160^-^ subset, which did not differ between unprimed and primed NK cells upon iEC, iBP, and IL-12/IL-18 stimulation (Figures 5B and 5C). This effect was specific to BP, as priming with THP-1+iEC did not induce a change in CD160 expression and heighted IFN-γ secretion upon re-stimulation was associated with the CD160^-^ subset only (Figure 5C). Overall, these results indicate that the induction of BP-specific memory-like features in human primary NK cells is restricted to a subset of CD160^+^ NK cells.

### Enrichment of CD160^+^ NK cells in recovered melioidosis patients

**Figure 6:**
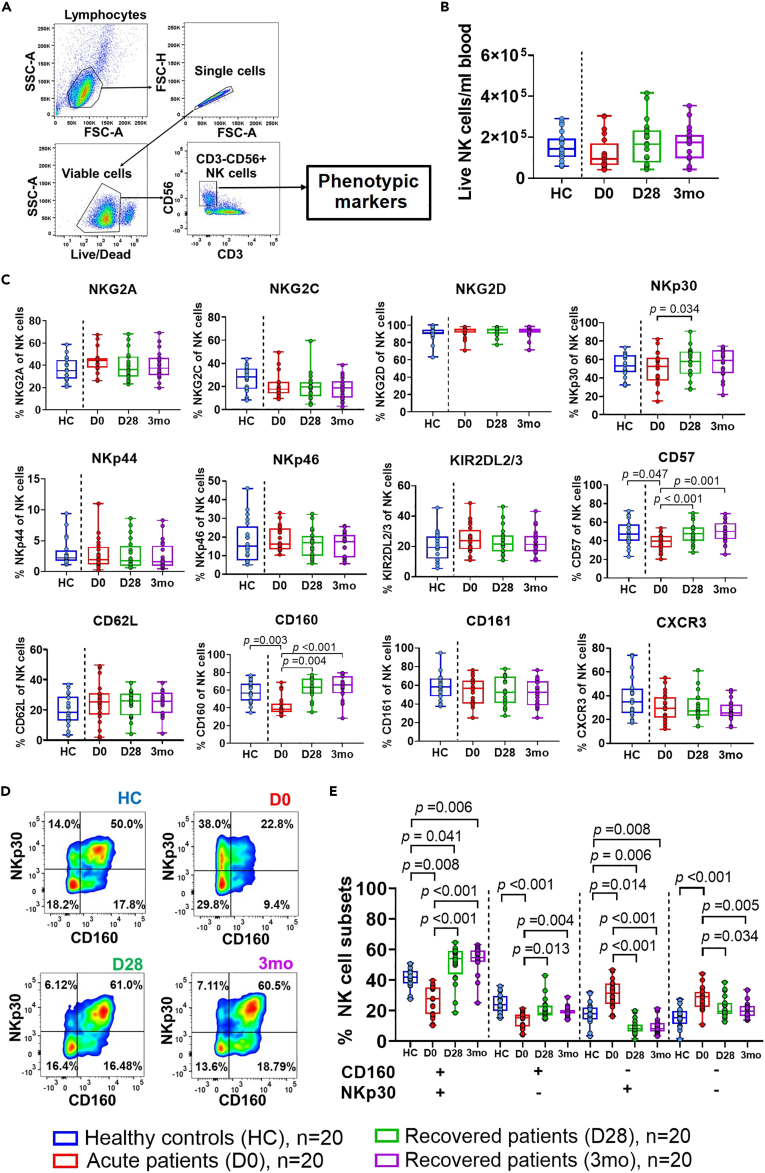
CD160^+^ NK cell subset co-expressing NKp30^+^ is enriched in recovered melioidosis patients PBMC from healthy donors (HC, n = 20), patients with acute melioidosis (D0), and recovered melioidosis patients at 28-day (D28, n = 20) and 3-month (3 months, n = 20) follow-up were used to determine NK cell characteristics by flow cytometry. (A) Gating strategy shown in representative flow cytometry dot blots. Lymphocytes (FSC-A, SSC-A) were gated followed by a single cell gate (FSC-A, FSC-H) and a viable cell gate (Live/Dead neg/lo). Bulk NK cells were identified as CD56^+^ and CD3^−^ within the single, viable cell gate and expression of NK cell phenotypic markers was identified within this gate. (B) The absolute frequency of NK cells in blood (cells/mL blood) and (C) expression of specific NK cell markers was determined on circulating NK cells in healthy donors and patients with melioidosis (acute, recovered). (D) Flow cytometry density plots of CD160 and NKp30 co-expression on circulating NK cells of one representative healthy donor and one paient with melioidosis during acute disease (day 0, D0) and upon recovery (day 28, D28 and 3 months, 3months). (E) The percentage of NK cells co-expressing CD160 and NKp30 in healthy donors and patients with melioidosis (acute, recovered). Phenotypic data are shown in box and whiskers plots. Statistical significance (p < 0.05) between multiple independent groups (healthy donors and melioidosis patients) was determined using Kruskal-Wallis test, followed by with Dunn’s method with the Benjamini-Hochberg method for multiple comparison. Friedman’s test, followed by Kruskal-Wallis, followed by with Dunn’s method with the Benjamini-Hochberg method for multiple comparison was used to compare statistical differences between multiple dependent groups (acute and recovered melioidosis patients). Only p values for statistically significant comparisons are shown on graphs.

We then sought to recapitulate our *in vitro* findings in a clinically relevant setting. To this end, we first determined the frequency and phenotype of circulating NK cells from healthy donors and patients with melioidosis from Northeast Thailand who did not have a previous history of melioidosis infection. Patients with acute melioidosis (Day 0) showed a trend toward a decrease in the absolute frequency of NK cells compared to the same cohort of recovered melioidosis patients (Day 28 and 3 months) (Figure 6B). Further phenotypic characterization of NK cells revealed a significant drop in CD160 and CD57-expressing NK cells in acute disease which increased upon recovery (28-day and 3-month follow-up) (Figure 6C). Recovered melioidosis patients showed higher frequency of NKp30-expressing NK cells at 28-day follow-up, and a trend toward increased frequency at 3-month follow-up, compared to patients with acute disease (Figure 6D). Other NK cell markers including NKG2A, NKG2C, NKG2D, NKp44, NKp46, KIR2DL2/3, CD161, CD62L, and CXCR3 remained unchanged (Figure 6D).

We next identified patterns of surface receptor co-expression on NK cells by looking at combinations of all the above markers. The only changes we observed were in CD160 and NKp30 co-expression with higher frequency of circulating CD160^+^NKp30^+^ NK cells present in recovered patients at 28-day and 3-month follow-up, compared to acute patients and healthy donors (p < 0.05 for all comparisons) (Figure 6E).

### CD160 expression on NK cells from recovered melioidosis patients correlates to memory-like properties

**Figure 7:**
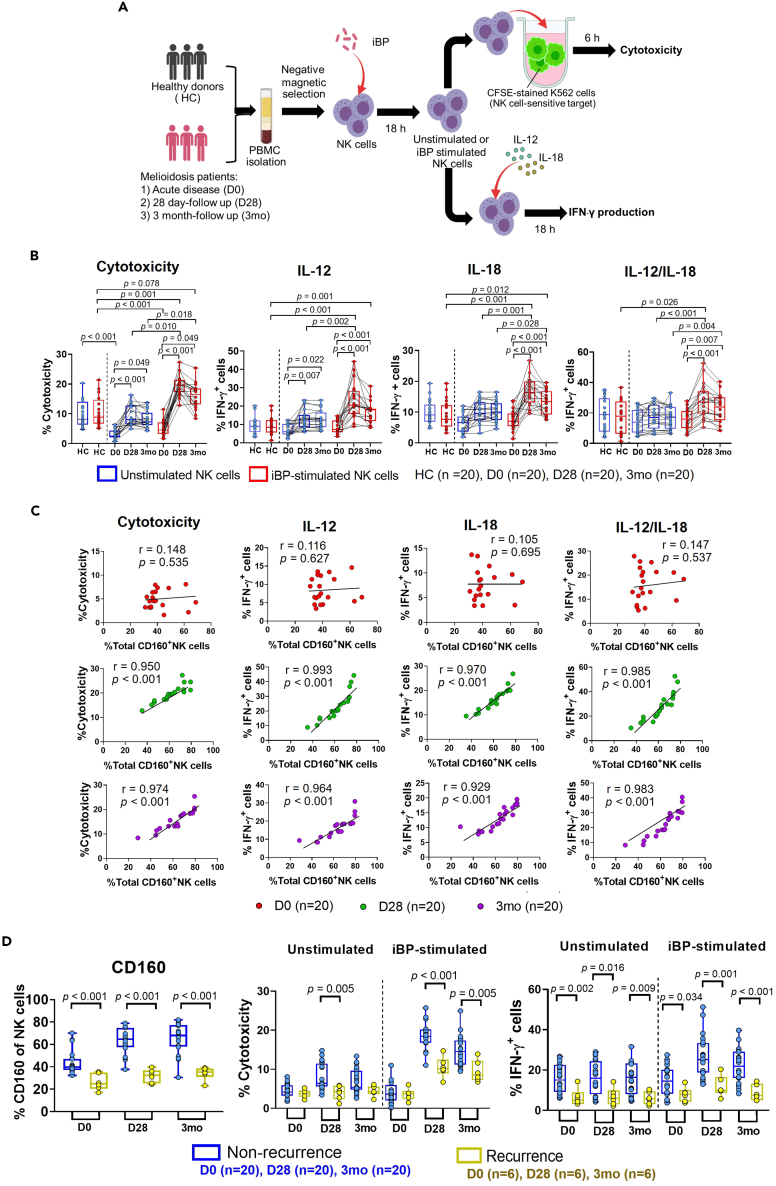
CD160 expression on NK cells from recovered melioidosis patients correlates with memory-like responses upon BP re-stimulation and is reduced in patients with a history of recurrent infection NK cells were isolated from PBMC of healthy endemic controls (HC) and patients with melioidosis with acute disease (D0) and 28-day (D28) and 3-month (3 months) follow-up. NK cells were incubated in the absence or presence of inactivated (heat-killed) BP (iBP) for 18 h. (A) Unstimulated or iBP-stimulated NK cells were either co-cultured with CFSE-stained K562 cells for assessment of cytotoxicity or stimulated with IL-12, IL-18, or IL-12/IL-18 for analysis of IFN-γ-producing NK cells (schematic workflow created with BioRender.com). (B) The percentage of NK cell cytotoxicity and relative frequency of IFN-γ-producing NK cells in healthy donors and patients with melioidosis at D0 (n = 20), D28 (n = 20), and 3 months (n = 20) were determined by flow cytometry. Statistical significance between multiple independent groups (healthy donors and melioidosis patients) was determined using Kruskal-Wallis test, followed by with Dunn’s method with the Benjamini-Hochberg method for multiple comparison. Friedman’s test, followed by with Dunn’s method with the Benjamini-Hochberg method for multiple comparison was used to compare statistical differences between multiple dependent groups (acute and recovered melioidosis patients). (C) Spearman’s correlation between NK cell responses upon iBP stimulation and relative frequency of total CD160-expressing NK cells. Individual dot plots represent patients at D0 (n = 20, red), D28 (n = 20, green), and 3 months (n = 20, purple). Spearman r and p values are shown on top of each graph. (B) and (C) represent data from patients with a first-time diagnosis of melioidosis. (D) The percentage of NK cell cytotoxicity and relative frequency of IL-12/IL-18-induced IFN-γ-producing NK cells in individuals with a first-time diagnosis of melioidosis (non-recurrent infection patients, n = 20) and individuals who had experienced one or more previous episodes of melioidosis (recurrent infection patients, n = 6) during acute disease (D0) and follow-up (D28, 3 months). Mann–Whitney U test was performed comparing recurrent and non-recurrent groups. Only p values of statistically significant comparisons (p < 0.05) are shown on graphs. The number of biological replicates is given in brackets at the bottom of each graph.

To establish whether NK cell memory is generated upon encounter with BP *in vivo*, we next examined NK cell function including cytotoxicity and cytokine-induced IFN-γ secretion upon BP re-exposure *ex vivo* (Figure 7A). Firstly, we only looked at patients without a previous history of melioidosis to capture *de novo* memory-like NK cell responses. NK cell function in the absence of iBP stimulation was reduced in patients with acute melioidosis, but this recovered over time with cytotoxicity and IL-12-induced IFN-γ secretion significantly higher at 28-day and 3-month follow-up (Figure 7B). iBP stimulation resulted in marked augmentation of NK cell function with a median increase of 19.5% (IQR 17.4 to 21.4%) in cytotoxicity, 20.5% (IQR 15.7 to 28.3%) in IL-12-induced IFN-γ production, 16.4% (IQR 13.2 to 19.9%) in IL-18-induced IFN-γ production, and 27.1% (IQR 19.5 to 34.3%) in IL-12/IL-18-induced IFN-γ production at 28-day follow-up, compared to unstimulated NK cells (Figure 7B). These responses remained elevated at the 3-month follow-up time point (Figure 7B). Importantly, there was no difference in NK cell function in healthy controls in presence or absence of iBP stimulation. Similar to our *in vitro* findings (Figure 3), CD160 expression alone strongly correlated with memory-like NK cell function (Spearman’s r > 0.9 and p < 0.001 for all correlations) (Figure 7C). This further strengthens the suitability of CD160 as a surface marker to track memory-like NK cells in melioidosis.

We then asked whether individuals who had a first-time diagnosis of melioidosis (non-recurrent infection, n = 20) show differences in quantity and quality of memory-like NK cell responses compared to individuals who had suffered one or more previous episodes of infection after completion of antibiotic treatment (recurrent infection, n = 6). There were no differences in the total frequency of circulating NK cells (Figure S6A) between the two groups and similar to non-recurrent individuals (Figure 7C), cytotoxicity and cytokine-induced IFN-γ production upon re-stimulation with iBP *ex vivo* strongly correlated with CD160 expression at 28-day and 3-month follow-up in individuals with recurrent infection (Figure S6B). However, the relative frequency of CD160^+^ NK cells and the magnitude of NK cell memory-like responses were significantly lower in recurrent patients (Figure 7D). Using a multivariable regression model, we show that CD160 expression on less than or equal to 37.1% (25^th^ percentile of non-recurrent melioidosis patients) NK cells is an independent correlate of recurrent melioidosis infection, when controlling for age, sex, BMI, diabetes status, and pre-existing liver and renal disease (adjusted Odds ratio 1.67 (95% CI 1.20–3.46), p = 0.0447; Table S2).

## Discussion

NK cells play a critical role in the elimination of infected host cells, thus providing an essential innate defense mechanism contributing to the early reduction of microbial burden. A growing body of evidence now suggests that NK cells are capable of memory responses to pathogens with implications for the design of novel therapeutics and vaccines. Here, we present a novel assay to study NK cell memory responses *in vitro* and demonstrate that priming with antigen-activated THP-1 cells is necessary for NK cells (both human cell line and primary cells) to acquire cell-intrinsic memory-like properties. Using this *in vitro* assay, we show phenotypic alterations, antigen-specific responses upon re-stimulation, as well as enhanced killing capacity of intracellular bacteria in NK cells primed with the Gram-negative intracellular pathogen BP, the causative agent of melioidosis.

Our data indicate that iBP-primed THP-1 cells can confer functional alterations in NK cells akin to memory-like properties with elevated levels of IFN-γ secretion in response to IL-12 and IL-18 stimulation. The presence of THP-1 cells was critical for generation of memory-like NK cells as IFN-γ levels were not different from unprimed NK cells when THP-1 cells or iBP alone were used for priming NK cells. THP-1 cells primed with inactivated bacteria highly upregulated IL-12 and IL-18 secretion. Both cytokines are known to induce IFN-γ production from NK cells, with dependence upon STAT4 phosphorylation^31^^,^^32^ and human NK cells can exhibit enhanced IFN-γ production after re-stimulation with IL-12 and IL-18.^26^ The biological activities of IL-12 and IL-18 are mediated via receptors composed of IL-12Rβ2 and IL-18Rɑ subunits, respectively, both of which are expressed on NK cells.^33^^,^^34^ We show that the strong IFN-γ response upon IL-12 and IL-18 stimulation of THP-1+iBP-primed NK cells is associated with increased T-bet, IL-12Rβ2, and IL-18Rɑ expression. This is in accordance with a report of memory-like NK cells in pleural fluid from patients with TB exhibiting high levels of T-bet expression.^35^ T-bet cooperates with the transcription factor Runx3 to control expression of IFN-γ, GzmB, and Pfr.^36^ Others have previously shown that epigenetic re-programming of the *IRF8* gene, an important regulator for the clonal expansion of MCMV-specific memory NK cells, is regulated by IL-12 signaling and the transcription factor STAT4.^37^

Memory-like NK cells can be characterized by enhanced responsiveness, such as increased cytotoxicity or cytokine expression upon re-stimulation. We show that BP-primed THP-1 cells induce memory-like NK cells with heightened bacteria-specific polyfunctional responses and killing capacity upon re-stimulation with bacterial antigens *in vitro*. This agrees with a previous report of human memory-like BCG-specific CD45RO^+^ NK cells producing more IFN-γ when exposed to BCG in the presence of monocytes than CD45RO^−^ NK cells.^18^^,^^35^ Another study illustrated that human memory-like NKG2C^+^ NK cells enhance IFN-γ production in response to CMV antigen in the presence of K-562 target cell lines.^38^ Memory-like NK cells from patients with advanced melanoma displayed enhanced IFN-γ production and cytotoxicity.^27^ In addition, Ly49H^+^ MCMV-specific memory NK cells exhibited more robust cytotoxic responses, higher amounts of IFN-γ compared to naive NK cells, and provide protective immunity following subsequent infection.^8^^,^^39^

It is known that memory-like NK cells generate a range of distinct phenotypic populations, which are specific to different microbial pathogens. We show a strong correlation of BP-induced memory-like NK cell function with expression of the surface marker CD160 *in vitro* and this is bacteria specific as heightened function is not associated with CD160 expression upon priming with *E. coli*. Furthermore, the induction of memory-like NK cells to BP *in vitro* is partly dependent on CD160 and IL-12Rβ2 indicating that both molecules contribute to memory-like differentiation of NK cells. Importantly, CD160 expression also strongly correlates with memory-like function in circulating NK cells from recovered melioidosis patients. At one and three months post acute disease, NK cells show heightened IFN-γ response and cytotoxicity when re-exposed to BP antigens *in vitro.* We further demonstrate that CD160 expression on NK cells in recovered patients is a correlate of repeated melioidosis infection with lower levels of this CD160^+^ memory-like NK cell population being associated with a history of recurrent melioidosis.

In humans, CD45RO^+^ memory-like NK cells from the pleural fluid of patients with TB have an important role in host response to *M. tuberculosis*, while peripheral blood CD56^dim^ NKp46^low^ NK cells show enhanced effector responses after influenza vaccination.^18^^,^^35^^,^^40^^,^^41^ Particular NK cell populations may be specifically activated to recognize distinct pathogens through their receptors, resulting in avidity selection of NK cells and a shift in the receptor repertoire to increase the frequency of specific receptor-defined sub-populations with memory functions.^42^ This is also supported by evidence that HCMV infection is associated with dramatic epigenetic imprinting on the NK cell repertoire.^43^ The mechanism by which NK cells specifically recognize a wide range of antigens is a matter of debate. There is some evidence that NK cell receptors might recognize antigens to mediate NK cell recall responses.^44^ Furthermore, NK cells are able to interact with bacteria directly. NK cells from healthy donors respond to BCG stimulation by upregulating activation molecules and enhancing cytokine production, irrespective of monocytes and macrophages.^45^^,^^46^ Recent studies have suggested that NK cells recognize bacterial antigens through the activating receptors NKp44 and TLR2.^28^^,^^46^ *M. tuberculosis*-infected macrophages can also be recognized via the NKp46-activating receptor.^27^

CD160 is a glycosylphosphatidylinositol-anchored Ig domain protein expressed on NK cells, intestinal intraepithelial lymphocytes, and γδ T cells.^47^ CD160 has been identified as a physiological ligand of MHC class I^48^ and this interaction has been shown to induce PI3K/Akt signaling in NK cells.^49^ PI3K/Akt signaling pathway plays a critical role in the differentiation of memory cells.^50^ In addition, CD160 expression has been negatively associated with histone-modification enzyme gene histone deacetylase 11 expression^51^ which associates with the *Tbet* gene promoter regions and plays a central role in memory development.^52^ Thus, it is plausible that engagement of CD160 through antigen: MHC complex can drive memory-like development in melioidosis.

Similar to a previous study showing that NK cells with highest avidity for MCMV antigen are preferentially selected to expand and comprise the memory NK cell population,^42^ we speculate that diversity in CD160 expression might drive NK cell functional heterogeneity during BP infection, with high-avidity NK cells being selected to dominate memory-like responses. BP antigen-specific NK cell receptors and avidity selection during melioidosis will be the focus of future studies.

Our findings also illustrate that NK cells have reduced CD160 expression, low cytotoxicity, and IFN-γ responses during acute disease, irrespective of BP antigen stimulation. This is in line with our previous work demonstrating that CD160 downregulation and functional impairment of NK cells were linked to fatality in acute melioidosis, indicating that NK cells are an immune correlate of protection.^21^^,^^53^ CD160 downregulation induced by plasma cytokines may result in impaired NK cell function during acute melioidosis. CD160 plays a role in NK-mediated IFN-γ secretion^54^ and decreased CD160 expression on NK cells can be attributed to high levels of tumor growth factor-β in acute melioidosis.^55^^,^^56^ In addition, it is possible that the specific engagement of CD160 induces tumor necrosis factor alpha, IL-6, and IL-8 cytokine production from NK cells.^57^ In this study, IFN-γ production was only focused as it has been considered as one of immune correlates of survival from melioidosis and for early control of BP infection in animal and clinical studies.^58^^,^^59^

Finally, we show that people with recurrent melioidosis infection have lower frequency of circulating CD160-expressing NK cells and reduced memory-like NK cell function compared to those with a first-time diagnosis of melioidosis giving further evidence for the importance of NK cells in protection from melioidosis. More studies specifically looking at a larger cohort of individuals with a history of recurrent melioidosis infection as well as detailed assessment of BP-specific memory-like NK cells in seropositive endemic individuals without a previous history of melioidosis infection are needed to further dissect the importance of this CD160^+^ NK cell subset in protection.

In conclusion, we demonstrate the suitability of a novel *in vitro* assay to interrogate functional and phenotypic properties of bacteria-induced memory-like NK cells, provide evidence of memory-like NK cell formation in melioidosis, and identify CD160 as a novel marker of BP-specific memory-like NK cells *in vitro* and *in vivo*. This will enable future monitoring of NK cell memory as a correlate of protection in vaccine studies and natural infection and will facilitate in-depth studies targeting this specific NK cell population using single-cell RNA sequencing and metabolomics.

### Limitations of the study

There are some limitations to our study, which are worth noting. Firstly, we were limited regarding the number of patients with melioidosis due to loss to follow-up and feasibility of recruitment. Secondly, availability of residual blood products to isolate primary NK cells for the development of our *in vitro* NK memory assay in Thailand was limited. Hence, the *in vitro* assay was established using the human NK-92 MI cell line and validated in primary human cells in the UK. Thirdly, replication for other bacterial infections is needed to confirm the suitability of CD160 as a marker for NK cell memory across different diseases. Fourthly, although our data suggest association between CD160 expression and NK cell memory in patients with melioidosis, it does not directly prove causality and this warrants further in-depth analysis and functional experiments on the CD160^+^ subset in a melioidosis cohort. Finally, approximately 77% of analyzed patients had diabetes and the non-diabetic group was too small to allow statistical comparison. Experimental evaluation of how this may affect the immune response in these donors should be further investigated.

## STAR★Methods

### Key resources table

**Table:** 

REAGENT or RESOURCE	SOURCE	IDENTIFIER
**Antibodies**		

Recombinant Rabbit IgG, monoclonal isotype control antibody (Clone: EPR25A)	Abcam	Cat#ab172730; RRID:AB_2687931
Purified Mouse IgG1, κ isotype control antibody (Clone: MG1-45)	Biolegend	Cat#401402; RRID:AB_2801451
Purified Mouse IgG2a, κ isotype control antibody (Clone: MOPC-173)	Biolegend	Cat#400202; RRID:AB_2927399
Purified Mouse IgG2b, κ isotype control antibody (Clone: eBMG2b)	Thermo Fisher Scientific	Cat#14-4732-81; RRID:AB_470116
Recombinant rabbit anti-human CD160 (Clone: EPR8054)	Abcam	Cat#ab128954; RRID:AB_11140414
Purified mouse anti-human NKp44 (Clone: P44-8)	Biolegend	Cat#325102; RRID:AB_756094
Purified mouse anti-human NKp46 (Clone 9E2)	Biolegend	Cat#331902; RRID:AB_1027637
Purified mouse anti-human IL-12Rβ (Clone: S16020B)	Biolegend	Cat#394202; RRID:AB_2734483
Purified mouse anti-human IL-15Rα (Clone: eBioJM7A4)	Thermo Fisher Scientific	Cat#14-7159-82; RRID:AB_657878
Purified mouse anti-human IL-18Rα (Clone: H44)	Biolegend	Cat#313804; RRID:AB_345312
BV711 mouse anti-human CD3 (Clone: OKT3)	Biolegend	Cat#317328; RRID:AB_2562907
AlexaFlour488 mouse anti-human CD16 (Clone: 3G8)	Biolegend	Cat#302019; RRID:AB_492974
FITC mouse anti-human CD25 (Clone: M-A251)	Biolegend	Cat#356106; RRID:AB_2561863
PE CD56 mouse anti-human CD56 (Clone: My31)	BD Bioscience	Cat#347747; RRID:AB_400346
BV605 mouse anti-human CD56 (Clone: HCD56)	Biolegend	Cat#318334; RRID:AB_2561912
PE mouse anti-human CD57 (Clone: HNK-1)	Biolegend	Cat#359612; RRID:AB_2562759
BV421 mouse anti-human CD62L (Clone: DREG-56)	Biolegend	Cat#304828; RRID:AB_2562914
APC mouse anti-human CD69 (Clone: L78)	BD Bioscience	Cat#340560; RRID:AB_400523
PE-Cy5 mouse anti-human CD107a (Clone: H4A3)	BD Bioscience	Cat#555802; RRID:AB_396136
FITC mouse anti-human CD158b/KIR2DL2/L3, NKAT2 (Clone: DX27)	Biolegend	Cat#312603; RRID:AB_314934
AlexaFlour488 mouse anti-human CD160 (Clone: BY155)	BD Bioscience	Cat#562351; RRID:AB_11153688
PE-Cy7 mouse anti-human CD161 (Clone: HP-3G10)	Biolegend	Cat#339918; RID:AB_11126745
FITC mouse anti-human CD183 (CXCR3)(Clone: G025H7)	Biolegend	Cat#353704; RRID:AB_10983066
APC mouse anti-human CD197(CCR7) (Clone: G043H7)	Biolegend	Cat#353214; RRID:AB_10917387
FITC mouse anti-human CD218a (IL-18Rα) (Clone: H44)	Biolegend	Cat#313810; RRID:AB_2123648
AlexaFlour647 mouse anti-human/mouse Granzyme B (Clone: GB11)	Biolegend	Cat#515406; RRID:AB_2566333
AlexaFlour647 mouse anti-human IL-12Rβ2 (Clone: S16020B)	Biolegend	Cat#394208; RRID:AB_2894595
PE mouse anti-human IFN-γ (Clone: 4S.B3)	Biolegend	Cat#502509; RRID:AB_315234
APC mouse anti-human NKG2A (Clone: S19005E)	Biolegend	Cat#375108; RRID:AB_2888862
PE mouse anti-human NKG2C (Clone: S19005E)	Biolegend	Cat#375004; RRID:AB_2888871
PE mouse anti-human NKG2D (Clone: 1D11)	Biolegend	Cat#320806; RRID:AB_492960
APC mouse anti-human NKp30 (Clone: P30-15)	Biolegend	Cat#325210; RRID:AB_2149449
PerCP/Cy5.5 mouse anti-human NKp30 (Clone: P30-15)	Biolegend	Cat#325216; RRID:AB_2716095
PE mouse anti-human NKp44 (Clone: P44-8)	Biolegend	Cat#325108; RRID:AB_756100
Pacific blue mouse anti-human NKp46 (Clone: 9E2)	Biolegend	Cat#331912; RRID:AB_2149280
PE mouse anti-human NKp46 (Clone: 29A1.4)	Biolegend	Cat#137604; RRID:AB_2235755
APC mouse anti-human Perforin (Clone: dG9)	Biolegend	Cat#308112; RRID:AB_2252843
PE mouse anti-human T-bet (Clone: 4B10)	BD Bioscience	Cat#561265; RRID:AB_10565980
PerCP mouse anti-human CD3 (Clone: UCHT1)	Biolegend	Cat#300428; RRID:AB_893298
Purified Mouse IgG1, κ isotype control antibody (Clone: MG1-45)	Biolegend	Cat#401402; RRID:AB_2801451
Purified Mouse IgG2a, κ isotype control antibody (Clone: MOPC-173)	Biolegend	Cat#400202; RRID:AB_2927399
Purified Mouse IgG2b, κ isotype control antibody (Clone: eBMG2b)	Thermo Fisher Scientific	Cat#14-4732-81; RRID:AB_470116
Recombinant rabbit anti-human CD160 (Clone: EPR8054)	Abcam	Cat#ab128954; RRID:AB_11140414
Purified mouse anti-human NKp44 (Clone: P44-8)	Biolegend	Cat#325102; RRID:AB_756094
Purified mouse anti-human NKp46 (Clone 9E2)	Biolegend	Cat#331902; RRID:AB_1027637
Purified mouse anti-human IL-12Rβ (Clone: S16020B)	Biolegend	Cat#394202; RRID:AB_2734483
Purified mouse anti-human IL-15Rα (Clone: eBioJM7A4)	Thermo Fisher Scientific	Cat#14-7159-82; RRID:AB_657878
Purified mouse anti-human IL-18Rα (Clone: H44)	Biolegend	Cat#313804; RRID:AB_345312
BV711 mouse anti-human CD3 (Clone: OKT3)	Biolegend	Cat#317328; RRID:AB_2562907
AlexaFlour488 mouse anti-human CD16 (Clone: 3G8)	Biolegend	Cat#302019; RRID:AB_492974
FITC mouse anti-human CD25 (Clone: M-A251)	Biolegend	Cat#356106; RRID:AB_2561863
PE CD56 mouse anti-human CD56 (Clone: My31)	BD Bioscience	Cat#347747; RRID:AB_400346
BV605 mouse anti-human CD56 (Clone: HCD56)	Biolegend	Cat#318334; RRID:AB_2561912
PE mouse anti-human CD57 (Clone: HNK-1)	Biolegend	Cat#359612; RRID:AB_2562759
BV421 mouse anti-human CD62L (Clone: DREG-56)	Biolegend	Cat#304828; RRID:AB_2562914
APC mouse anti-human CD69 (Clone: L78)	BD Bioscience	Cat#340560; RRID:AB_400523
PE-Cy5 mouse anti-human CD107a (Clone: H4A3)	BD Bioscience	Cat#555802; RRID:AB_396136
FITC mouse anti-human CD158b/KIR2DL2/L3, NKAT2 (Clone: DX27)	Biolegend	Cat#312603; RRID:AB_314934
AlexaFlour488 mouse anti-human CD160 (Clone: BY155)	BD Bioscience	Cat#562351; RRID:AB_11153688
PE-Cy7 mouse anti-human CD161 (Clone: HP-3G10)	Biolegend	Cat#339918; RID:AB_11126745
FITC mouse anti-human CD183 (CXCR3)(Clone: G025H7)	Biolegend	Cat#353704; RRID:AB_10983066
APC mouse anti-human CD197(CCR7) (Clone: G043H7)	Biolegend	Cat#353214; RRID:AB_10917387
FITC mouse anti-human CD218a (IL-18Rα) (Clone: H44)	Biolegend	Cat#313810; RRID:AB_2123648
AlexaFlour647 mouse anti-human/mouse Granzyme B (Clone: GB11)	Biolegend	Cat#515406; RRID:AB_2566333
AlexaFlour647 mouse anti-human IL-12Rβ2 (Clone: S16020B)	Biolegend	Cat#394208; RRID:AB_2894595
PE mouse anti-human IFN-γ (Clone: 4S.B3)	Biolegend	Cat#502509; RRID:AB_315234
APC mouse anti-human NKG2A (Clone: S19005E)	Biolegend	Cat#375108; RRID:AB_2888862
PE mouse anti-human NKG2C (Clone: S19005E)	Biolegend	Cat#375004; RRID:AB_2888871
PE mouse anti-human NKG2D (Clone: 1D11)	Biolegend	Cat#320806; RRID:AB_492960
APC mouse anti-human NKp30 (Clone: P30-15)	Biolegend	Cat#325210; RRID:AB_2149449
PerCP/Cy5.5 mouse anti-human NKp30 (Clone: P30-15)	Biolegend	Cat#325216; RRID:AB_2716095
PE mouse anti-human NKp44 (Clone: P44-8)	Biolegend	Cat#325108; RRID:AB_756100
Pacific blue mouse anti-human NKp46 (Clone: 9E2)	Biolegend	Cat#331912; RRID:AB_2149280
PE mouse anti-human NKp46 (Clone: 29A1.4)	Biolegend	Cat#137604; RRID:AB_2235755
APC mouse anti-human Perforin (Clone: dG9)	Biolegend	Cat#308112; RRID:AB_2252843
PE mouse anti-human T-bet (Clone: 4B10)	BD Bioscience	Cat#561265; AB_10565980

**Bacterial and virus strains**		

*B.thailandensis* E264	Narisara Chantratita	N/A
*B.pseudomallei* K96243	Narisara Chantratita	N/A
*E.coli* DH5α	Narisara Chantratita	N/A

**Biological samples**		

PBMC from healthy individuals and melioidosis patients	Mukdahan Hospital in Mukdahan and Mahidol University, Thailand	N/A
PBMC from healthy individuals	University of Oxford	N/A

**Chemicals, peptides, and recombinant proteins**		

RPMI-1640 Medium with L-Glutamine	Gibco	Cat#11875093
MEM-α medium, no nucleosides	Gibco	Cat#12561056
Fetal Bovine Serum	Hyclone	Cat#SH30080.03
Horse Serum	Gibco	Cat#16050122
0.25% Trypsin-EDTA	Gibco	Cat#25200072
GlutaMax Supplement	Gibco	Cat#35050061
Penicillin/Streptomycin	Gibco	Cat#15140122
Kamanycin	Gibco	Cat#15160054
Folic acid	Sigma	Cat#8758-5G
Myo-inositol	Sigma	Cat#I7508-50G
2-Mercaptoethanol	Gibco	Cat#21985023
DPBS without calcium, magnesium	Hyclone	Cat#SH30028.01
NK MACS Medium	Miltenyi Biotec	Cat#130-114-429
Penicillin/Streptomycin	Sigma	Cat#P0781
L-Glutamine	Sigma	Cat#G7513
Human AB Serum	Sigma	Cat#H4522-100ML
Recombinant Human IL-2	Biolegend	Cat#589102
Recombinant Human IL-15	Biolegend	Cat#570302
Phosphate bufferred saline tablets	Oxoid	Cat#BR0014G
Columbia Blood Agar Base	Oxoid	Cat#CM0331
Lymphoprep	STEMCELL Technology	Cat#07861
7-AAD Viability Staining Solution	Thermo Fisher Scientific	Cat#00-6993-50
Zombie NIR Fixable Viability Dye	Biolegend	Cat#423105
PMA	Sigma	Cat#P1585-1MG
Ionomycin calcium salt from *Streptomyces conglobatus*	Sigma	Cat#I0634-1MG
Brefeldin A	Biolegend	Cat#420601
Golgi Stop Protein Transport Inhibitor containing monensin	BD Bioscience	Cat#554724
Perm/Wash Buffer	BD Bioscience	Cat#554723
Fetal Bovine Serum, heat inactivated, sterile filtered	Himedia	Cat#RM9955-500ML
Carboxyfluorescein succinimidyl ester (CFSE)	BD Bioscience	Cat#565082
TritonX-100	Sigma	T9284-100ML
Paraformaldehyde	Merk Millipore	Cat#818715
Human FcR Blocking Reagent	Miltenyi Biotec	Cat#130-059-901
Sodium azide	Sigma	Cat#S2002-25G
Bovine Serum Albumin (BSA)	Sigma	Cat#A7906-100G

**Critical commercial assays**		

Human IL-12(p70) ELISA Set	BD Bioscience	Cat#555183
Human IL-15 ELISA Set	BD Bioscience	Cat#559268
Human IFN-γ ELISA Set	BD Bioscience	Cat#555142
Human IL-18 ELISA Kit	Abcam	Cat#224877
EasySep Human NK Cell Isolation Kit	STEMCELL Technology	Cat#17955

**Experimental models: Cell lines**		

A549 cell line	ATCC	Cat#CCL-185
K562 cell line	ATCC	Cat#CRL-243
THP cell line	ATCC	Cat#TIB-202
NK-92 MI cell line	ATCC	Cat#CRL-2408

**Software and algorithms**		

Flowjo 10.8.1	BD Bioscience	https://www.flowjo.com/
Prism 9.0	GraphPad	https://www.graphpad.com/

**Other**		

BD FACSAria III Flow cytometer	BD Bioscience	N/A
MACSQuant X Flow cytometer	Miltenyi Biotec	N/A

### Resource availability

#### Lead contact

Further information and requests for resources and reagents should be directed to and will be fulfilled by the lead contact, Narisara Chantratita (narisara@tropmedres.ac).

#### Material availability

This study did not generate new unique reagents.

### Experimental model and subject details

#### Cell lines and culture conditions

The human NK cell line, NK-92MI (ATCC CRL-2408) was cultured in complete MEM-α (cMEM-α): minimum essential medium-α (MEM-α) without nucleosides and supplemented with 2.2 g/L sodium bicarbonate, 2 mM L-glutamine, 100 U/ml penicillin plus 100 μg/ml streptomycin (Gibco, CA, USA), 0.02 mM folic acid (Sigma, St. Louis, MO, USA), 0.2 mM myo-inositol (Sigma), 0.1 mM 2-mercaptoethanol (Sigma), 12.5% FBS (Hyclone) and 12.5 % horse serum (Gibco) in a humidified incubator with 5% CO_2_ at 37°C. Adenocarcinomic human alveolar basal epithelial cells (A549, ATCC CCL-185), human monocytic leukemia cell line (THP-1, ATCC TIB-202) and human erythroleukemic cell line (K562, ATCC CRL-243) were cultured in R10 media, known as RPMI 1640 (Gibco) supplemented with 10% heat-inactivated fetal bovine serum (FBS) (Himedia, Mumbai, India), 100 U/ml penicillin, 100 μg/ml streptomycin ( Gibco) and 2 mM GLutaMAX (Gibco), at 37°C with 5% CO_2_.

#### Study design and subjects

A longitudinal study was conducted in healthy donors (n=30) and melioidosis patients (n=26) at Mukdahan Hospital, Mukdahan, Northeast Thailand. Inclusion criteria for the melioidosis cohort were Thai male or female patients aged 18 years or older with *B. pseudomallei* culture-confirmed melioidosis from any clinical specimen taken within 24 h after admission to the hospital. Exclusion criteria were pregnancy, receiving palliative care or incarceration. *B. pseudomallei* was identified by biochemical tests and latex agglutination^60^ at the Microbiology laboratory of the hospital and further confirmed by Matrix-Assisted Laser Desorption Ionization Mass Spectrometry (MALDI-TOF MS) as previously described.^61^ Healthy donors were enrolled at the hospital’s blood donation clinic. Inclusion criteria for the healthy cohort were Thai male or female and ≥ 18 years of age. Exclusion criteria were pregnancy or delivery in the past nine months, weight of less than 40 kg or greater than 136 kg, previous history of melioidosis, recent illness, any chronic medical condition or medications and any organ failure (such as cirrhosis), any immune system deficiency, vaccination within the past six weeks, use of any immune-modifying agents,any anti-inflammatory medication or biological agents used for cell depletion in the past week, infectious symptoms in the past two weeks, vigorous exercise in the past 24 h, or alcohol use in the past 24 h.

Whole blood samples were obtained from healthy donors and melioidosis patients at the day of recruitment (defined as day 0). Blood samples were further collected from melioidosis patients at 28-day and 3-month follow-up time points. Recurrent melioidosis infection was defined as the subsequent episode of infection determined by clinical samples with *B. pseudomallei*-confirmed culture after completion of antibiotic treatment for the first episode.^62^ Clinical data were obtained by the study team or extracted from medical records. Mortality data was collected from hospital mortality records and follow-up phone calls for 28 days.

#### Ethic statement

The clinical study protocol was reviewed and approved by the Ethics committees of the Faculty of Tropical Medicine, Mahidol University (MUTM 2021-033-01), Mukdahan Hospital (MEC 07/64) and the University of Oxford (REC 21/YH/0206). The study was conducted according to the principles of the Declaration of Helsinki (2008), the International Conference on Harmonization (ICH) and Good Clinical Practice (GCP) guidelines. All volunteers provided written informed consent to participate in this project.

### Method details

#### PBMC isolation

Peripheral blood mononuclear cells (PBMC) were isolated from 20 ml of heparinized blood within 3 h of sample collection by using 50 ml Sepmate tubes as described by the manufacturer (STEMCELL Technologies, Canada). Briefly, blood was diluted 1:1 in R10 media prior to transfer to Sepmate tubes containing 15 ml of Lymphoprep (Axis Shiled, Oslo Norway). PBMC were washed twice with 25 ml of R10 media and then cryopreserved in FBS containing 10% dimethyl sulfoxide (DMSO) (Sigma, St. Louis, MO, USA) at –80°C and stored in liquid nitrogen until use.

#### Isolation and expansion of NK cells from PBMCs

Primary NK cells were isolated from at least 5 x 10^6^ cells of cryopreserved PBMCs using the EasySep Human NK cell Enrichment Kit according to the manufacturer’s instructions (STEMCELL Technologies, Canada). The purity of NK cells was > 95% of CD3^-^CD56^+^ cells as determined by flow cytometry. Primary NK cells obtained from healthy donors (n =4) were cultured and expanded in NK MACs medium (Miltenyi Biotec, Bergisch-Gladbach, Germany) supplemented with 10% human AB serum (Sigma,), 1 mM Penicillin/Streptomycin (Sigma), 5 ng/ml of rhIL-2 and rhIL-15 (Biolegend, CA, USA) at 37°C with 5% CO_2_ for 7-10 days. Expanded NK cells were used in the experimental validation of the NK cell memory assay.

#### Preparation of inactivated bacteria

Bacterial culture was performed in a BSL-3 laboratory. *B. pseudomallei* K96243 (BP), *B. thailandensis* E264 (BT) and *E. coli* DH5α (EC) were grown on Columbia blood agar (Oxoid, UK) and incubated at 37°C for 16–18 h. The bacteria were harvested with a loop, suspended and washed twice in 1 ml of PBS. Bacteria were re-suspended at 10^9^ CFU/ml in PBS and heated at 80°C for 1 h or fixed in 0.5% paraformaldehyde. Inactivated bacteria were washed three times with PBS and cell viability was verified by colony count on Columbia agar. The bacterial suspensions were stored at -80°C until use.

#### Flow cytometry

Cells were washed twice with PBS containing 0.1% BSA and 0.05% sodium azide (NaN_3_) (FACS buffer). For surface staining, cells were incubated with live/dead fixable stain and fluorochrome-conjugated primary human-specific monoclonal antibodies (mAbs) in the presence of human FcR blocking reagent (Miltenyi Biotec) at 4°C for 30 min in the dark. Cells were washed twice with FACS buffer and then fixed in 2% paraformaldehyde. For intracellular staining (ICS), Brefeldin A (BD Biosciences Franklin Lakes, NJ, USA) was added at final dilution of 1: 1000 to the cells 4 h before measurement. The cells were then washed and fixed with fixation/permeabilization solution (BD Biosciences) at 4°C for 20 min, washed with permeabilization buffer (BD Biosciences) followed by incubation with fluorochrome-conjugated mAbs in the presence of human FcR blocking reagent at 4°C for 30 min in the dark. After washing with permeabilization buffer, the samples were resuspended in FACS staining buffer and acquired immediately or stored at 4°C in the dark for up to 24 h prior to acquisition. Primary NK cells from healthy individuals used for validation of the *in vitro* memory NK cell assay were acquired on a MACS Quant X (Miltenyi Biotec). All other samples were acquired on a BD FACAriaIII (BD Biosciences). Data analysis was performed using FlowJo software, version 10 (BD Biosciences). Details of monoclonal antibodies (mAbs) labeled with fluorochromes are listed in Table S3.

#### NK cell memory assay

THP-1 cells resuspended in 1 ml of R10 were plated at 1 × 10^6^ live cells/well into 24-well flat bottom plates. The cells were stimulated with inactivated BP (iBP) or inactivated *E. coli* (iEC) at a multiplicity of infection per cell (MOI) of 4-100 at 37°C with 5% CO_2_ for 24 h. To prime NK-92 MI cells, the cells at 1.5 × 10^6^ live cells resuspended in R10 were co-cultured with iBP-primed THP-1 cells (THP-1+iBP or THP-1 + iEC) at 37°C with 5% CO_2_ for 24 h. NK-92 MI cells: THP-1+iBP or THP-1+iEC ratio is 1.5 for co-culture. Primed NK-92 MI cells were purified using the EasySep human NK cell enrichment kit (STEMCELL Technologies). Primed NK cells were resuspended in 1ml of cMEM-α media and plated at  5× 10^5^ live cells/well in a 24-well flat bottom plate at 37°C, 5% CO_2_ for 7 days and then used in further experiments.

For experimental validation, primary NK cells resuspended in R10 were co-cultured with THP-1 + iBP (MOI 100) or THP-1 + iEC (MOI 100) at 37°C, 5% CO_2_ for 24 h. Primed NK cells were purified by magnetic negative isolation. The cells resuspended in NK MACs medium (Miltenyi Biotec) supplemented with with 10% human AB serum (Sigma), 1 mM Penicillin/Streptomycin (Sigma), 5 ng/ml of rhIL-2 and rhIL-15 (Biolegend) were plated at 3.5× 10^5^ live cells/well into 24-well flat bottom plates at 37°C, 5% CO_2_ for 7 days and then used in further experiments. Culture medium was refreshed on day 4.

#### Measurement of THP-1 cell-derived cytokines

THP-1 cells resuspended in 1 ml of R10 were plated at 1 × 10^6^ live cells/well into 24-well flat bottom plates and then stimulated with LPS derived from *E. coli* (1 μg/ml, Sigma) or iBP at MOI 4, 20 or 100 at 37°C with 5% CO_2_ for 24 h. Cell-free supernatant was collected by centrifugation at 1,000× g for 5 min, and 12,000× g for 5 min. IL-12, IL-15 and IL-18 secretion was measured by Human IL-12 (p70), Human IL-15 ELISA Set (BD Biosciences) and Human IL-18 ELISA kit (Abcam, Canbridge, UK) according to the manufacturer’s instructions.

#### Cytokine stimulation assay

Unprimed or primed NK-92 MI cells resuspended in 500μl of cMEM-α media were seeded at 5×10^5^ live cells/well into 24-well tissue culture plates. The cells were stimulated with 5 ng/ml of rhIL-12, rhIL-15 and rhIL-18 (Biolegend) alone or in combination for 18 h. Phorbol myristic acetate (PMA) (20 ng/ml; Sigma) plus ionomycin (1 μg/ml; Sigma) were used as positive control. Cell-free culture supernatant was collected by centrifugation at 1,000× g for 5 min and measured for IFN-γ production by Human IFN-γ ELISA Set (BD Biosciences) according to the manufacturer’s instructions. For *in vitro* re-stimulation, primary NK cells obtained from a clinical cohort were resuspended in R10 and then plated at 1.25×10^5^ live cells/well into 96-well round-bottom culture plate. The cells were incubated in the absence or presence of iBP (MOI 100) for 18 h. Unstimulated or stimulated NK cells were subsequently stimulated with 5 ng/ml of rhIL-12 and rhIL-18 (Biolegend CA, USA) for 18 h. NK cells were analysed for IFN-γ production using flow cytometry.

#### Polyfunctionality assay

Unprimed or primed NK-92 MI cells were plated at 5×10^5^ live cells/well into a 24-well cell culture plate,and stimulated with inactivated bacteria (including iBP, iBT and iEC) at MOI 100, NK-cell sensitive target cells (K562 cells), THP-1 cells, or THP-1 cells previously primed with inactivated bacteria at 37°C, 5% CO_2_ for 18 h. For the last 4 h, CD107a PE-Cy5 (BD Biosciences), Brefeldin A (Biolegend) and Golgi Stop containing monensin (BD Biosciences) were added to cultures at a final concentration of 0.5 μg/ml, 10 μg/ml and 0.16 μM, respectively. Cells were subsequently subjected to intracellular staining and flow cytometry as described above.

For blocking surface and cytokine receptors of NK cells, the following anti-human monoclonal antibodies were used: CD160 (clone EPR8054, Abcam), NKp44 (clone P44-8), NKp46 (clone 9E2), IL-12Rβ2 (clone S16020B), IL-18Rα (clone H44) (all Biolegend) and IL-15α (clone eBioJM7A4, Thermo Fisher Scientific, US). Rabbit IgG-(clone EPR25A) (Abcam), mouse IgG1-(clone MG1-45), mouse IgG2a-(clone MOPC-173) (Biolegend) and mouse IgG2b- (clone eBMG2b) (Thermo Fisher Scientific) mAbs were used as isotype control. NK-92 MI cells were treated with isotype or blocking antibodies at a final concentration of 10 μg/ml at 37°C, 5% CO_2_ for 30 min and subsequently primed with iBP-primed THP-1 cells or THP-1 alone for 24 h. After negative isolation and 7 day-cultivation, primed NK cells were then re-stimulated with iBP and functional responses were assessed as described above.

#### Measurement of intracellular bacterial killing and infected cell death

A549 or THP-1 cells resuspended in R10 media were used as target cells (T) and seeded at 1×10^6^ live cells/well into 96-well round-bottom culture plates and incubated at 37°C, 5% CO_2_ overnight. Cells were washed with PBS and infected with BP at MOI 100 for 2 h. Infected cells were washed with PBS and further incubated in R10 media containing 250 mg/ml of kanamycin (Gibco) at 37°C, 5% CO_2_ for 2 h. Unprimed or primed NK-92 MI cells were used as effector cells (E) and were incubated with iBP (MOI 100) at 37°C,5% CO_2_ for 24 h. Infected A549 or THP-1 cells were washed with PBS and then co-cultured with effector cells at E: T ratios of 1, 3 or 10 and incubated at 37°C, 5% CO_2_ for 2 h. For measuring intracellular bacterial killing, infected cell culture was centrifuged at 125× g for 10 min, then washed and lysed with 0.1% Triton X-100. The cell lysate was plated on Columbia blood agar (Oxoid) and incubated overnight at 37°C. The viability of intracellular bacteria was colony counted and presented as colony-forming unit/ml (cfu/ml). For measuring infected host cell death, the infected cell culture was centrifuged at 125× g for 10 min, then washed with PBS and incubated with live/dead fixable Near IR dye (Biolegend) and a PE-conjugated CD56 mAb (clone My31, BD Bioscience) at a dilution of 1:1000 and 1: 100, respectively. The percentage of infected host cells were determined by flow cytometry. Infected host cells were defined as CD56 ^-^ and live/dead Near IR^+^ cells.

#### Cytotoxicity assay

Primary NK cells that were incubated in the absence or presence of iBP (MOI 100) at 37°C, 5% CO_2_ for 18 h were used as effector cells (E). K562 cells labeled with carboxyfluorescein succinimidyl ester (CFSE) (BD Biosciences) at a final concentration of 2 μM were used as target cells (T). CFSE-labeled K562 cells were washed twice before the killing assay. Subsequently, unstimulated or iBP stimulated NK effector cells resuspended in R10 media were plated at 1.5×10^5^ live cells/well and incubated with target cells at an E: T ratio of 1 in a 96 well-round bottom plate. After co-culture at 37°C, 5% CO_2_ for 6 h, the cell mixture was washed twice with PBS and then stained with viability dye 7-AAD (Thermo Fisher Scientific, USA) at a dilution of 1:25 for 15 min. The percentage of NK cell cytotoxicity was analysed by flow cytometry, and calculated per well using the following equation:

$$NK\;cell\;cytotoxicity\;\left(\%\right)=\frac{\left(\%\;experimental\;cell\;lysis-\%\;spontaneous\;cell\;lysis\right)}{\left(\%\;maximum\;cell\;lysis-\%\;spontaneous\;cell\;lysis\right)}\times 100$$

where % experimental cell lysis is the percentage of 7-AAD^+^ CFSE^+^ K562 cells (dead cells) when co-cultured with NK cells, the % spontaneous cell lysis is the percentage of 7-AAD^+^ CFSE^+^ K562 cells in the absence of effector cells and % maximum cell lysis is the percentage of 7-AAD^+^ CFSE^+^ K562 cells harvested from wells containing 0.1% Triton X-100.

### Quantification and statistical analysis

Statistical analysis was performed using Prism software version 9.0 (GraphPad Software Inc, La Jolla, CA, USA). Specifications of tests exploited and sample size for each experiment are mentioned in the figure legends. For the differences between multiple independent groups, a non-parametric Kruskal-Wallis test, followed by Dunn’s method with the Benjamini-Hochberg method for multiple comparison was applied for the cell line experiments and the assessment of the surface protein markers of interest and NK cell responses in healthy donors and melioidosis patients at different time points (day 0, 28 day- and 3 month-follow up). A non-parametric Friedman’s test, followed by subsequent Dunn’s method with the Benjamini-Hochberg method for multiple comparison was used to test for multiple dependent group differences between melioidosis patients during acute disease (day 0) and long-term follow up (28 days and 3 months). Statistical differences between recurrent and non-recurrent groups were analyzed by Mann–Whitney U-test for nonparametric data. A two-tailed Wilcoxon matched-pairs signed-rank test was performed to compare statistical differences between unstimulated and iBP-stimulated NK cells in melioidosis patients. Correlation was performed via Spearman’s rank correlation coefficient. To test the association of phenotypic parameters with outcome (recurrent infection), we performed univariable and multivariable logistic regression adjusting for age, sex, BMI, diabetes status, preexisting liver and renal disease. CD160 expression on NK cells (CD160^+^ percentage of NK cells) was analyzed as categorical variable by assigning each subject to one of two groups using the 25% percentile of the non-recurrent group as cut-off. In all analyses, statistical significance was considered as *p* < 0.05 and all tests were two-tailed.

## Data Availability

• Data reported in this paper will be shared by the lead contact upon request. • This paper does not report original code. • Any additional information required to reanalyze the data reported in this work paper is available from the lead contact upon request.
