# Supplementary material for: Identification and function of a novel human memory-like NK cell population expressing CD160 in melioidosis
Source: iScience. 2023 Jun 28;26(8):107234. doi: 10.1016/j.isci.2023.107234 (PMC10372747; doi:10.1016/j.isci.2023.107234)
Supplement: Document S1. Figures S1–S7 and Tables S1–S3 [file mmc1.pdf]

iScience, Volume *26*

Supplemental information

Identification and function of a novel human memory-like NK cell population expressing CD160 in melioidosis

Anucha Preechanukul, Barbara Kronsteiner, Natnaree Saiprom, Kitilak Rochaikun, Boonthanom Moonmueangsan, Rungnapa Phunpang, Orawan Ottiwet, Yuphin Kongphrai, Soonthon Wapee, Kesinee Chotivanich, Chumpol Morakot, Rachan Janon, Susanna J. Dunachie, and Narisara Chantratita

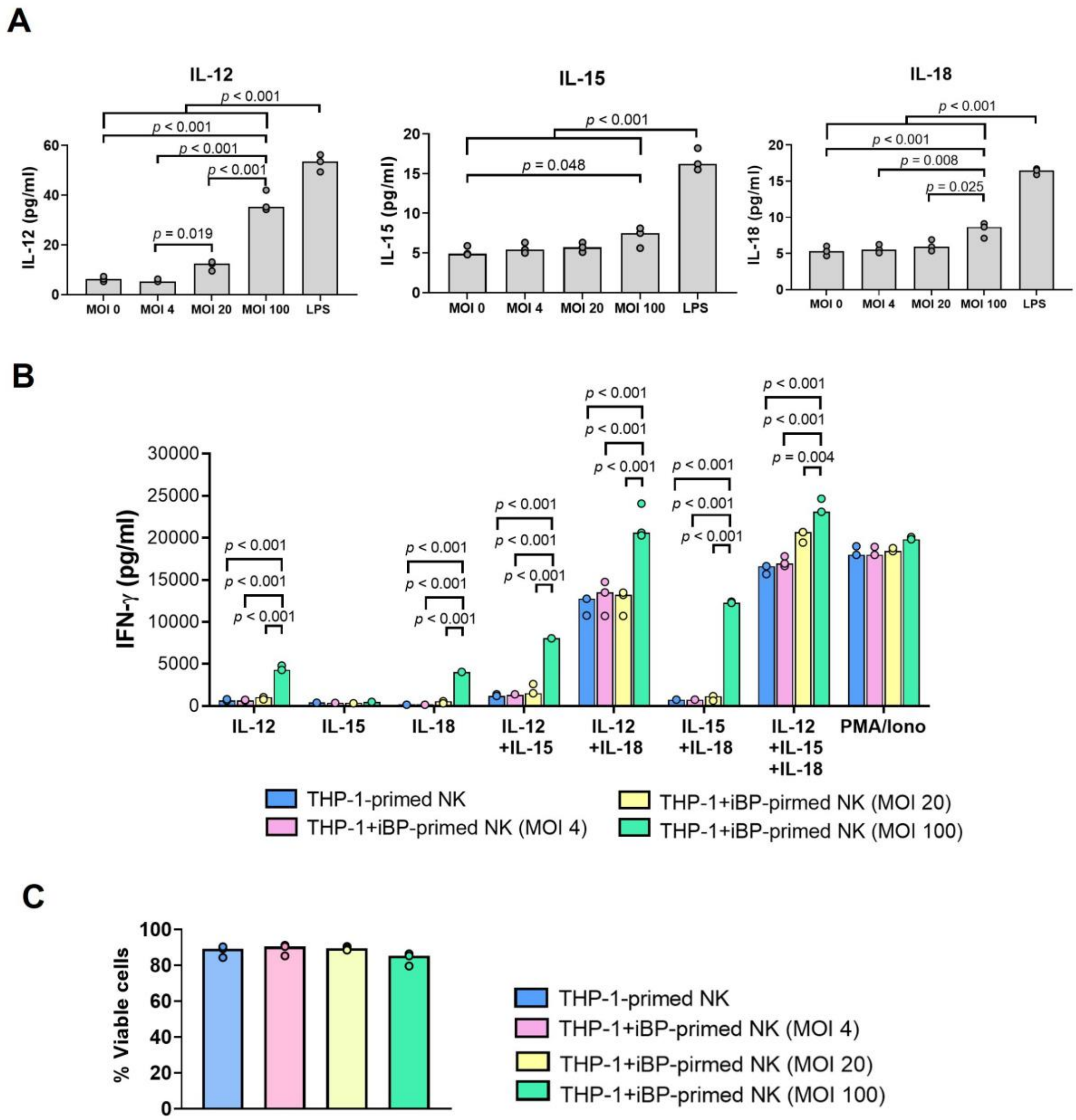

Figure S1. Cytokine production profile of iBP-primed THP-1 cells and THP-1+iBP-primed NK-92 MI cells, related to Figure 1. (A) The level of IL-12, IL-15 and IL-18 secretion by unstimulated THP-1 cells and THP-1 stimulated with *E. coli* LPS (1 µg/ml) or inactivated (heat-killed) *B. pseudomallei* (iBP) at MOI 4, 20 or 100 for 24 h. The level of cytokine secretion in supernatants was evaluated by ELISA. **(B)** NK-92 MI cells were primed with THP-1 cells, or THP-1+iBP (MOI 4, 20 or 100) for 24 h, followed by negative enrichment and cultured for 7 days. Primed NK-92 MI cells were then stimulated with IL-12, IL-15, IL-18 or combinations thereof for 18 h and IFN-γ production was measured by ELISA. **(C)** The percentage of cell viability of NK-92 MI cells primed with THP-1 cells, or THP-1+iBP (MOI 4, 20 or 100) determined by flow cytometry. Three independent experiments were performed with three technical replicates each. The medians of the technical replicates were used for statistical testing and graphical presentation. Statistical differences were calculated using Kruskal-Wallis test, followed by

with Dunn’s method with the Benjamini-Hochberg method for multiple comparison. A p value of < 0.05 is considered statistically significant.

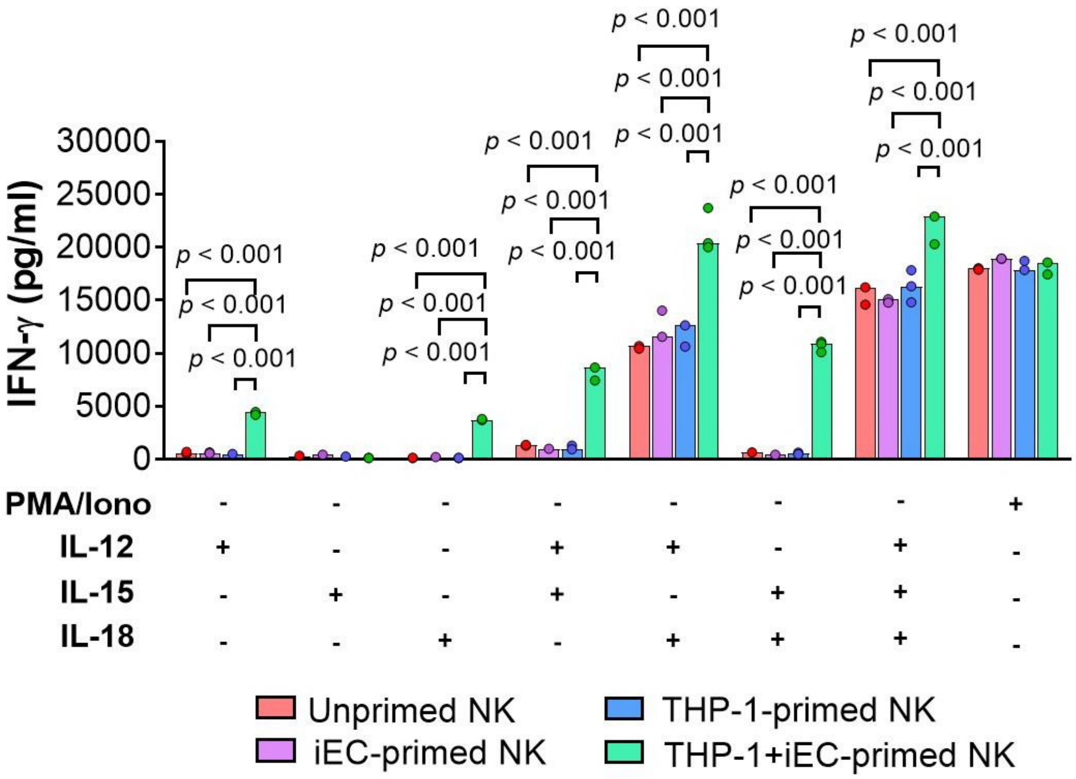

Figure S2. ***E. coli*- primed THP-1 cells potentiate IFN-γ secretion from NK-92 MI cells, related to Figure 1.** NK-92 MI cells were primed with THP-1 cells, or THP-1+inactivated (heat-killed) *E.coli* (iEC) for 24 h, followed by negative enrichment and culture for 7 days. Primed NK-92 MI cells were then stimulated with IL-12, IL-15, IL-18 or combinations thereof for 18 h and IFN-γ production was measured by ELISA. Three independent experiments were performed with three technical replicates each. The medians of the technical replicates were used for statistical testing and graphical presentation. Statistical differences were calculated using Kruskal-Wallis test, followed by with Dunn's method with the Benjamini-Hochberg method for multiple comparison. A p value of < 0.05 is considered statistically significant.

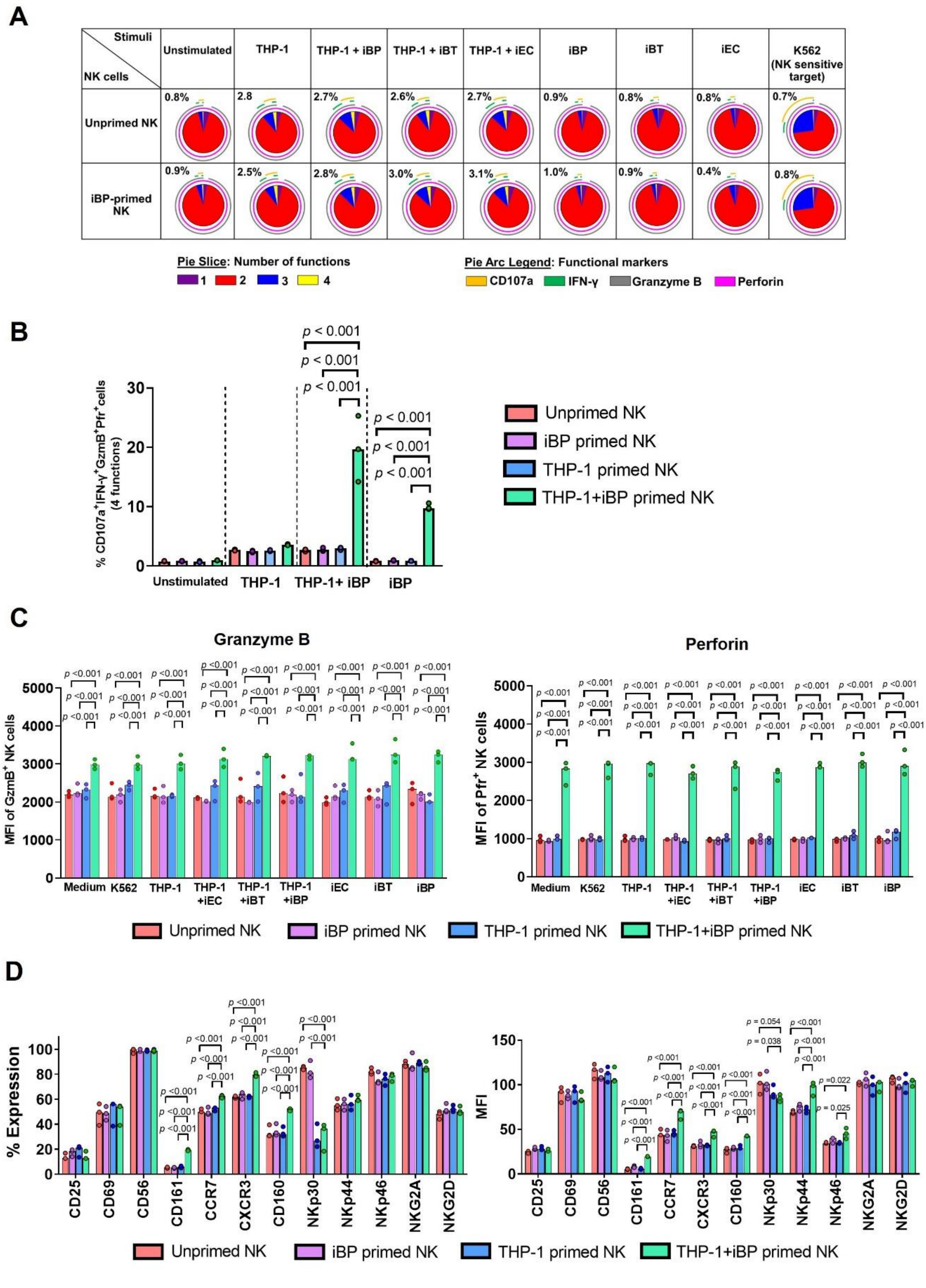
A
Stimuli
NK cells
Unstimulated
THP-1
THP-1 + iBP
THP-1 + iBT
THP-1 + iEC
iBP
iBT
iEC
K562 (NK sensitive target)
Unprimed NK
0.8%
2.8
2.7%
2.6%
2.7%
0.9%
0.8%
0.8%
0.7%
iBP-primed NK
0.9%
2.5%
2.8%
3.0%
3.1%
1.0%
0.9%
0.4%
0.8%
Pie Slice: Number of functions
1
2
3
4
Pie Arc Legend: Functional markers
CD107a
IFN-γ
Granzyme B
Perforin
B
% CD107a⁺IFN-γ⁺GzmB⁺Pfr⁺cells (4 functions)
Unstimulated
THP-1
THP-1+ iBP
iBP
p < 0.001
Unprimed NK
iBP primed NK
THP-1 primed NK
THP-1+iBP primed NK
C
Granzyme B
MFI of GzmB⁺ NK cells
Medium
K562
THP-1
THP-1 +iEC
THP-1 +iBT
THP-1 +iBP
iEC
iBT
iBP
Perforin
MFI of Pfr⁺ NK cells
p <0.001
Unprimed NK
iBP primed NK
THP-1 primed NK
THP-1+iBP primed NK
D
% Expression
MFI
CD25
CD69
CD56
CD161
CCR7
CXCR3
CD160
NKp30
NKp44
NKp46
NKG2A
NKG2D
p <0.001
p = 0.054
p = 0.038
p =0.022
p = 0.025
Unprimed NK
iBP primed NK
THP-1 primed NK
THP-1+iBP primed NK

Figure S3. Polyfunctionality profile of NK-92 MI cells primed with iBP and expression of surface as well as cytotoxicity markers on primed NK-92 MI cells, related to Figure 2. (A) NK-92 MI cells were primed in the absence or presence of inactivated (heat-killed) *B. pseudomallei* (iBP), cultured for 7 days and then stimulated with THP-1, THP-1+iBP, THP-1+inactivated *B. thailandensis* (iBT), THP-1+inactivated *E. coli* (iEC), iBP, iBT, iEC, K562 or remained unstimulated for 18 h. Polyfunctionality was assessed by flow cytometry and is presented in pie charts. Pie arcs represent proportions of NK cell responses to specific markers including CD107a (orange arcs), IFN-γ, (green arcs), granzyme B (gray arcs) and perforin (pink arcs). Pie slices represent percentages of NK-92 MI cells co-expressing 1 (violet), 2 (red), 3 (blue) or 4 (yellow) functional markers in responses to mentioned stimuli. The percentage of cells expressing all 4 functional markers are given above each pie chart. Data were summarized from three-independent experiments, which were performed with three technical replicates each. **(B)** The percentage of unprimed and primed NK-92 MI cell co-expressing CD107a, IFN-γ, granzyme B and perforin in response to different stimuli. **(C)** Median fluorescence intensity (MFI) of granzyme B (GzmB) and perforin (Pfn) expression on unprimed, iBP-primed, THP-1-primed, THP-1+iBP- primed NK-92 MI cells cultured for 7 days and stimulated with or without THP-1, THP-1+iBP, THP-1+iBT, THP-1+iEC, iBP, iBT, iEC, K562 for 18 h. **(D)** The percentage and MFI of surface markers on unprimed and primed NK-92 MI cells (7 days post priming). Three independent experiments were performed with three technical replicates each. The medians of the technical replicates were used for statistical testing and graphical presentation. Statistical differences were calculated using Kruskal-Wallis test, followed by with Dunn's method with the Benjamini-Hochberg method for multiple comparison. A p value of < 0.05 is considered statistically significant.

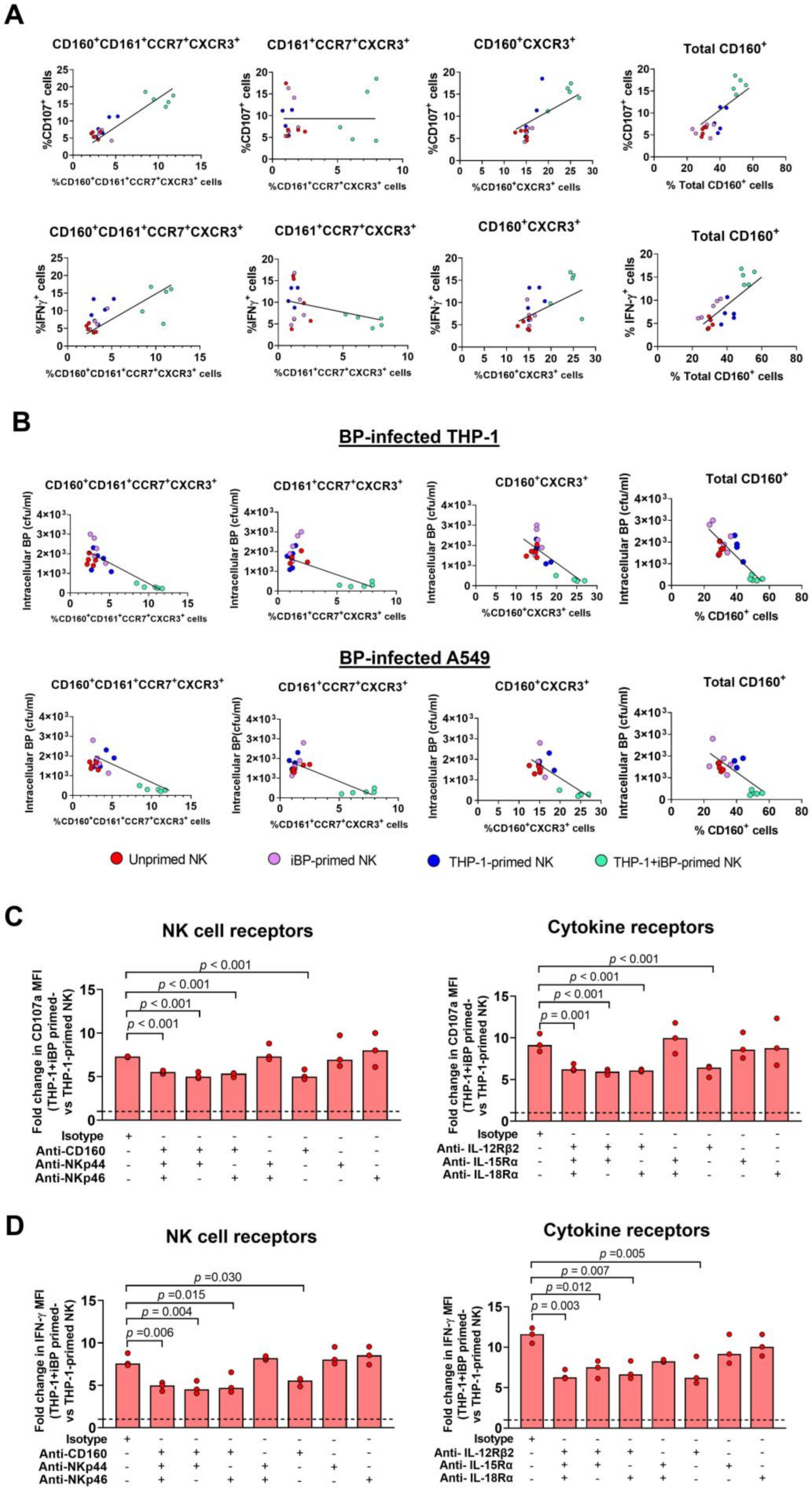
A
CD160+CD161+CCR7+CXCR3+
CD161+CCR7+CXCR3+
CD160+CXCR3+
Total CD160+
%CD107+ cells
%IFNγ+ cells
% IFN-γ+ cells
%CD160+CD161+CCR7+CXCR3+ cells
%CD161+CCR7+CXCR3+ cells
%CD160+CXCR3+ cells
% Total CD160+ cells
B
BP-infected THP-1
BP-infected A549
Intracellular BP (cfu/ml)
% CD160+ cells
Unprimed NK
iBP-primed NK
THP-1-primed NK
THP-1+iBP-primed NK
C
NK cell receptors
Cytokine receptors
Fold change in CD107a MFI (THP-1+iBP primed- vs THP-1-primed NK)
p < 0.001
p = 0.001
Isotype
Anti-CD160
Anti-NKp44
Anti-NKp46
Anti- IL-12Rβ2
Anti- IL-15Rα
Anti- IL-18Rα
D
NK cell receptors
Cytokine receptors
Fold change in IFN-γ MFI (THP-1+iBP primed- vs THP-1-primed NK)
p =0.030
p =0.015
p = 0.004
p =0.006
p =0.005
p = 0.007
p =0.012
p = 0.003

Figure S4. Comparison plot of phenotypic and functional characteristics of primed NK-92 MI cells and impact of surface and receptor blocking on generation of NK-92 MI cells with memory-like function, related to Figure 3 and 4. The frequency of four NK cell subsets exhibiting the following marker profile: CD160/CD161/CXCR3/CCR7, CD161/CXCR3/CCR7, CD160/CXCR3 and CD160 were compared with functional properties including **(A)** degranulation, IFN-γ production and **(B)** NK cell-mediated killing of intracellular BP by unprimed NK-92 MI cells and NK-92 MI cells primed with inactivated (heat-killed) *B. pseudomallei* (iBP), THP-1 cells or THP+iBP. Five-independent experiments were performed with three technical replicates each. **(C, D)** Effect of blocking surface receptors on the acquisition of memory-like NK cell function. NK-92 MI cells were treated with mAbs against CD160, NKp44, NKp46, IL-12Rβ2, IL-15Rα and IL-18Rα and primed with THP-1 and THP-1+iBP for 24 h. NK-92 MI cells were negatively enriched and culture for 7 days followed by stimulation with iBP for 18 h. Degranulation (CD107a) and IFN-γ production were determined using flow cytometry. Three independent experiments were performed with three technical replicates each. The medians of the technical replicates were used for statistical testing and graphical presentation. Statistical differences were calculated using Kruskal-Wallis test, followed by with Dunn's method with the Benjamini-Hochberg method for multiple comparison. A *p* value of < 0.05 is considered statistically significant.

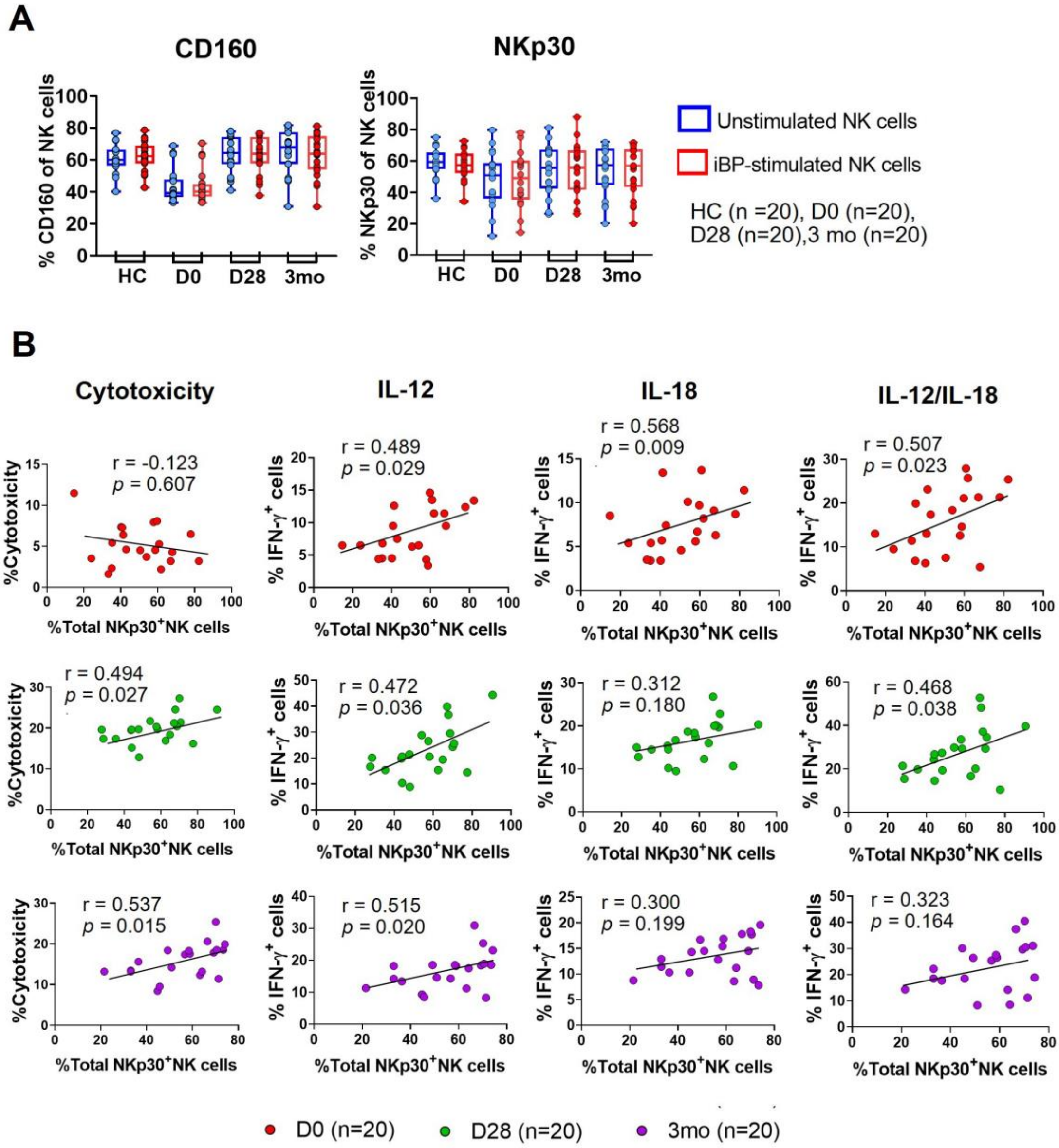

Figure S5. Impact of *in vitro* stimulation on CD160 and NKp30 expression on NK cells from melioidosis patients and correlation of NKp30 expressing NK cell frequency to NK cell function, related to Figure 7. NK cells were isolated from PBMC of from melioidosis patients with acute disease (D0, n =20), 28 day-(D28, n=20) and 3 month- (3mo, n=20) follow up. NK cells were incubated in the absence or presence of iBP for 18 h. **(A)** The percentage of CD160 and NKp30 expressing NK cells was determined by flow cytometry and presented in box and whiskers plots showing individual data points. Statistical analysis was performed using a two-tailed Wilcoxon matched-pairs signed-rank test. A *p* value of $p < 0.05$ was considered significant. No statistically significant differences were observed. **(B)** Spearman's correlation analysis was performed between NK cell responses (including cytotoxicity and IL-12, IL-18 or IL-12/IL-18 induced IFN-γ production) and the relative frequency of NKp30

expressing NK cells upon iBP stimulation. Individual dot plots represent patients at D0 (red), D28 (green) and 3mo (purple). Spearman r and *p* values are shown on top of each graph.

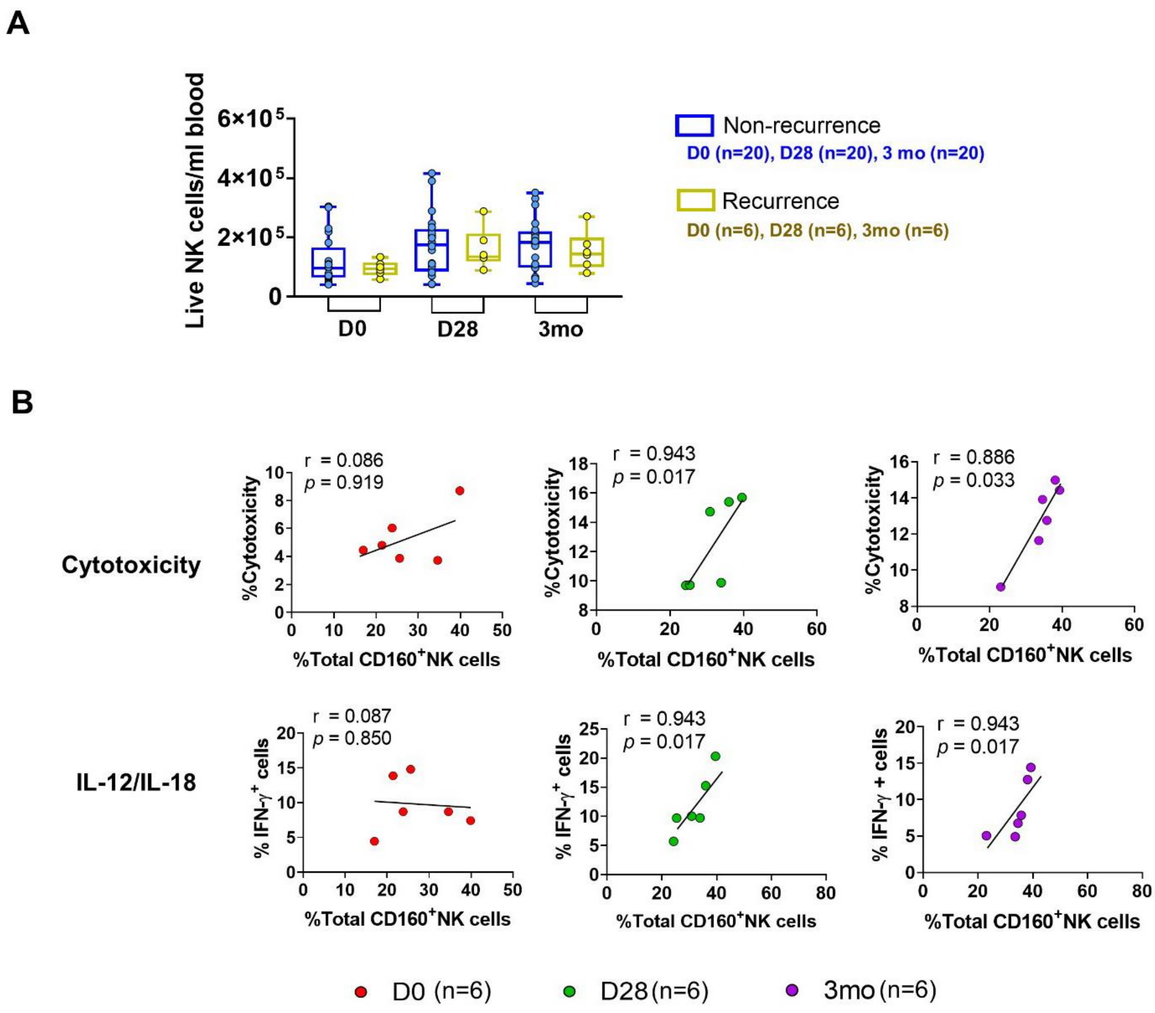

Figure S6. Memory-like NK cell responses in recurrent melioidosis patients, related to Figure 7. **(A)** The absolute frequency of NK cells (cells/ml blood) in individuals with a first time diagnosis of melioidosis (non-recurrent patients) and individuals who had experienced multiple episodes of melioidosis (recurrent patients) during acute disease (D0), 28 day-(D28,) and 3 month- (3mo) follow up was assessed by flow cytometry. Data are presented as box and whiskers plots showing individual data points. Statistical analysis was performed using Mann–Whitney U test and only *p* values for statistically significant ($p < 0.05$) comparisons are presented on the graphs**.** **(B)** Spearman's correlation analysis was performed between NK cell responses upon iBP stimulation (cytotoxicity and IL-12/IL-18-induced IFN-γ production) and the relative frequency of NK cells expressing CD160 in recurrent melioidosis patients. Spearman r and *p* values are shown on top of each graph

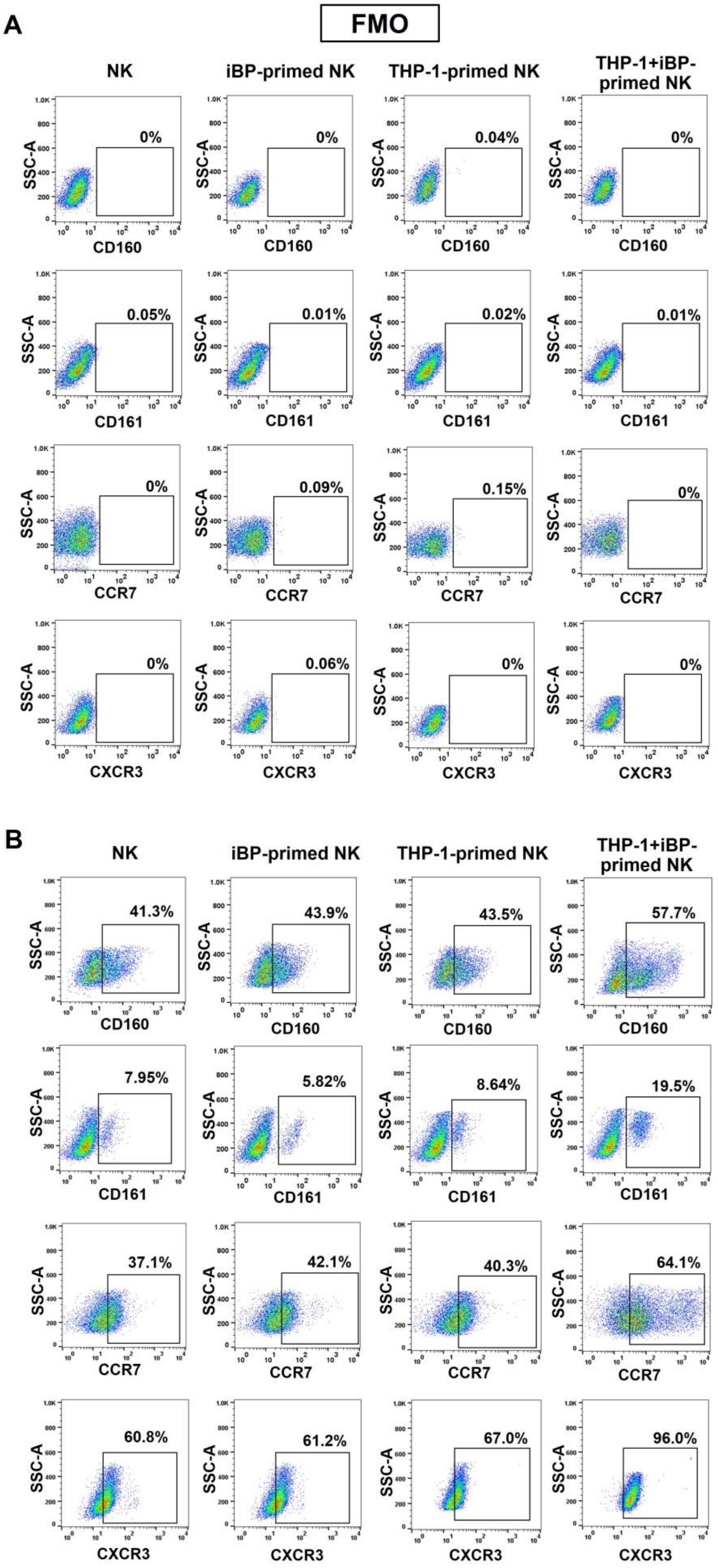
A
FMO
NK
iBP-primed NK
THP-1-primed NK
THP-1+iBP-primed NK
SSC-A
CD160
0%
0%
0.04%
0%
CD161
0.05%
0.01%
0.02%
0.01%
CCR7
0%
0.09%
0.15%
0%
CXCR3
0%
0.06%
0%
0%
B
NK
iBP-primed NK
THP-1-primed NK
THP-1+iBP-primed NK
CD160
41.3%
43.9%
43.5%
57.7%
CD161
7.95%
5.82%
8.64%
19.5%
CCR7
37.1%
42.1%
40.3%
64.1%
CXCR3
60.8%
61.2%
67.0%
96.0%

Figure S7. Representative flow cytometry pseudocolor dot plots for phenotypic characterization of NK-92 MI cells, related to Figure 3. NK-92 MI cells were primed with inactivated (heat-killed) *B. pseudomallei* (iBP), THP-1, THP-1+iBP or remained unprimed for 24 h, subsequently negatively enriched and cultured for 7 days. **(A)** Representative plots of fluorescence minus one (FMO) controls for CD160, CD161, CCR7 and CXCR3 against SSC-A in unprimed and primed NK-92 MI cells. **(B)** Representative plots of unprimed and primed NK-92 MI cells expressing CD160, CD161, CCR7 and CXCR3 against SSC-A.

Table S1. Demographic characteristics of melioidosis and healthy control cohort for phenotypic and functional analyses of NK cells, related to STAR METHODS (Experimental model and subject details)

Characteristics	Melioidosis patients (n=26)	Healthy donors (n=30)
Age in years, median (IQR)	53 (44-56)	42(37-48)
Sex Male/Female(M%)	19/7 (73.07)	12/18 (40)
Bacteremia (%)	17 (65.38)	N/A
Cardiovascular disease (%)	1 (3.85)	N/A
Pre-existing liver disease (%)	2(7.69)	N/A
Pre-existing renal disease (%)	2 (7.69)	N/A
Diabetes (%)	20 (76.92)	N/A
Hypertension (%)	4(15.38)	N/A
Pneumonia (%)	5(19.23)	N/A
Previous history of melioidosis	5 (19.23)	N/A
Recurrent melioidosis infection	6 (23.08)	N/A

Table S2. Uni- and multivariable analysis to determine correlates of recurrent infection in melioidosis patients, related to Figure 7D.

Variables	Recurrent melioidosis (n=6)	Non-recurrent melioidosis (n=20)	Univariable	Multivariable	
			***p*-value**	**Adjusted Odds Ratio (95% CI)**	***p*-value**
Age in years, median (IQR)	54 (47-56)	51(38-56)	0.9438	0.92(0.66-1.15)	0.5322
Sex (Male/Female)	4/2	15/5	0.2757	0.89(0.14-7.63)	0.9085
Body mass index (BMI) in kg/m^2, median (IQR)	20.59 (19.28-22.91)	21.11 (18.22-26.51)	0.6233	0.77(0.51-1.07)	0.1262
Diabetes	6	14	0.6996	0.67(0.095-5.86)	0.6876
Pre-existing liver disease	0	2	0.2754	0.71(0.057-16.80)	0.7909
Pre-existing renal disease	1	1	0.4579	0.33(0.012-9.20)	0.4611
$CD160^+$ percentage of NK cells (≤ 37.1%)[a]	6	5	0.0124	1.67(1.20-3.46)	0.0447

[a]Cutoff was set below the 25% percentile of non-recurrent melioidosis patients

Table S3. Reagents and fluorochrome-conjugated monoclonal antibodies (mAbs) for analyzing cell phenotype and function, related to STAR METHODS (Flow cytometry)

Markers	Fluorochrome /Reporter	Clone	Target species	Host species	Isotype	Manufacturer	Stock conc. (μg/ml)	Final conc. (μg/ml)	Optimal dilution	Characteristic measured
CD3	BV711	OKT3	Human	Mouse	IgG1, κ	Biolegend	40	0.4	1:100	Cell surface protein
CD3	PerCP	UCKT1	Human	Mouse	IgG1, κ	Biolegend	100	1	1:100	Cell surface protein
CD16	AF488	3G8	Human	Mouse	IgG1, κ	Biolegend	100	2	1:50	Cell surface protein
CD25	FITC	M-A251	Human	Mouse	IgG1, κ	Biolegend	50	1	1:50	Cell surface protein
CD56	PE	My31	Human	Mouse	IgG1, κ	BD Bioscience	125	1.25	1:100	Cell surface protein
CD56	BV605	HCD56	Human	Mouse	IgG1, κ	Biolegend	100	3.03	1:33	Cell surface protein
CD57	PE	HNK-1	Human	Mouse	IgG1, κ	Biolegend	25	0.167	1:150	Cell surface protein
CD62L	BV421	DREG-56	Human	Mouse	IgG1, κ	Biolegend	50	0.5	1:100	Cell surface protein
CD69	APC	L78	Human	Mouse	IgG1, κ	BD Bioscience	200	4	1:50	Cell surface protein
CD107a	PE-Cy5	H4A3	Human	Mouse	IgG1, κ	BD Bioscience	50	0.5	1:100	Cell surface protein
CD158b/KIR2DL2-3	FITC	DX27	Human	Mouse	IgG1, κ	Biolegend	400	12.12	1:33	Cell surface protein
CD160	AF488	BY155	Human	Mouse	IgG1, κ	BD Bioscience	100	3.33	1:30	Cell surface protein
CD160	PerCP-Cy5.5	7H1	Human	Mouse	IgG1, κ	Biolegend	200	6.67	1:30	Cell surface protein
CD161	PE-Cy7	HP-3G10	Human	Mouse	IgG1, κ	Biolegend	100	2	1:50	Cell surface protein
CD183 (CXCR3)	FITC	G025H7	Human	Mouse	IgG1, κ	Biolegend	200	4	1:50	Cell surface protein
CD197 (CCR7)	APC	G043H4	Human	Mouse	IgG1, κ	Biolegend	200	2	1:100	Cell surface protein
CD218a (IL-18Rα)	FITC	H44	Human	Mouse	IgG1, κ	Biolegend	100	2.5	1:40	Cell surface protein
Granzyme B	AF647	GB11	Human,Mouse	Mouse	IgG1, κ	Biolegend	20	0.25	1:80	Intracellular protein
IL-12Rβ2	AF647	S16020B	Human	Mouse	IgG1, κ	Biolegend	100	2.5	1:40	Cell surface protein
IFN-γ	PE	4S.B3	Human	Mouse	IgG1, κ	Biolegend	10	0.25	1:40	Intracellular protein
NKG2A	APC	S19004C	Human	Mouse	IgG1, κ	Biolegend	100	2	1:50	Cell surface protein
NKG2C	PE	S19005E	Human	Mouse	IgG1, κ	Biolegend	50	1	1:50	Cell surface protein
NKG2D	PE	1D11	Human	Mouse	IgG1, κ	Biolegend	200	2	1:100	Cell surface protein
NKp30	APC	P30-15	Human	Mouse	IgG1, κ	Biolegend	50	1.67	1:30	Cell surface protein
NKp30	PerCP/Cy5.5	P30-15	Human	Mouse	IgG1, κ	Biolegend	100	2	1:50	Cell surface protein
NKp44	PE	P44-8	Human	Mouse	IgG1, κ	Biolegend	100	2.5	1:40	Cell surface protein
NKp46	Pacific blue	9E2	Human	Mouse	IgG1, κ	Biolegend	500	12.5	1:40	Cell surface protein
NKp46	PE	29A1.4	Human	Mouse	IgG1, κ	Biolegend	100	2	1:50	Cell surface protein
Perforin	APC	dG9	Human	Mouse	IgG1, κ	Biolegend	50	0.625	1:80	Intracellular protein
T-bet	PE	4B10	Human	Mouse	IgG1, κ	Biolegend	25	0.625	1:40	Intracellular protein
Fixable Near IR Dead Cell	Near IR	NA	Human	Mouse	NA	Invitrogen	NA	NA	1:1000	Cell death
Viability dye	7-AAD	NA	Human	Mouse	NA	Invitrogen	NA	NA	1:1000	Cell death
